# Characterizing Hydrated Polymers via Dielectric Relaxation Spectroscopy: Connecting Relative Permittivity, State of Water, and Salt Transport Properties of Sulfonated Polysulfones

**DOI:** 10.1021/acs.macromol.5c00616

**Published:** 2025-07-25

**Authors:** Sean M. Bannon, Beatrice M. Tremblay, Andrew Boudreau, Nurshaun Sreedhar, Caroline Morin, Charles R. Leroux, Phu Phan, Abhishek Roy, Mou Paul, Geoffrey M. Geise

**Affiliations:** † Department of Chemical Engineering, University of Virginia, 385 McCormick Road, Charlottesville, Virginia 22903, United States; ‡ 53405 National Renewable Energy Laboratory, Golden, Colorado 80401, United States; § Department of Chemical Engineering, University of California Santa Barbara, Santa Barbara, California 93106, United States; ∥ Maseeh Department of Civil, Architectural and Environmental Engineering, 12330 The University of Texas at Austin, Austin, Texas, 78712-1700, United States

## Abstract

Sulfonated polysulfone is a promising membrane material for separation and energy generation processes that rely on membranes to control the rates of small-molecule (e.g., water and ions) transport. The interactions among water molecules, ions, and the sulfonate groups in these polymers play a key role in controlling these rates of transport, but much remains unknown about these fundamental interactions in sulfonated polymers. In this study, we used dielectric relaxation spectroscopy to characterize water molecule dynamics in sulfonated polysulfone and Nafion. We found that the charged sulfonate groups contribute to a restriction of water molecule dynamics (i.e., a reduction in the characteristic time scale of dipolar motions) in a manner that is governed by the concentration and nature (i.e., conjugate base strength) of the sulfonate group. Additionally, we develop strategies to use these data to aid in modeling ion transport in sulfonated polysulfone. These results may be useful to guide engineering strategies for polymeric membranes.

## Graphical abstract

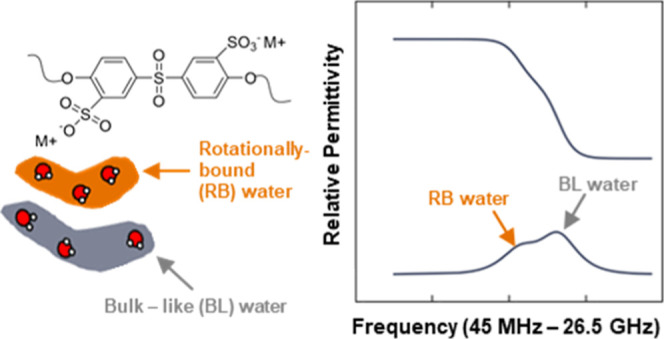

## 1 Introduction

Charge-containing polymers are of interest for use in a variety of energy generation and separation applications (e.g., fuel cells, reverse osmosis, and electrodialysis).^1–7^ In these applications, charged polymers are used as separation materials to selectively transport a species of interest while rejecting other components in the system (e.g., selectively transport water while rejecting salt in desalination applications).^8^ Despite considerable efforts to understand the molecular underpinnings that govern selective transport through charged polymers, there are still opportunities to understand how polymer chemistry relates to polymer transport properties to develop engineering strategies for next-generation polymeric membrane materials.

The solvent, ion, and proton transport properties of charged polymers often correlate strongly with the amount of water in the polymer matrix (i.e., water content).^8–12^ However, strong interactions between water molecules and the hydrophilic charged groups tethered to the polymer backbone can cause the properties of water molecules in the polymer matrix to be significantly different than the properties of water molecules in bulk solution.^10,12,13^ As a result, information about these water molecule properties and the state of water in the polymer often informs polymer transport properties to a greater extent than simply considering only the concentration of water molecules in the polymer.

Thermal techniques (e.g., differential scanning calorimetry (DSC)) have been used to characterize the state of water in charged polymers.^10,11,14^ In these techniques, different states of water are distinguished from each other based on the temperature where water molecules undergo phase transitions. For example, in DSC, water molecules in the polymer that freeze at 0 °C are considered free (or bulk-like) water that does not interact appreciably with the polymer matrix, and water molecules that freeze below 0 °C are considered freezable bound water because weak interactions between polymer and water molecules can depress the freezing point of water.^10^ Finally, many charged polymers contain significant populations of nonfreezable water, and these water molecules are those molecules that are so strongly bound to the polymer that they cannot participate in freezing processes.^10^

One issue associated with characterizing the state of water in charged polymers via thermal techniques is that to distinguish between different states of water, there must be a large enough concentration of bulk-like (or loosely bound) water in the polymer to observe the latent heat of the transition. Many charged polymers of interest for separation applications have low water content,^8^ and in these materials, a relatively large fraction of water molecules may be tightly bound to the hydrophilic charged functional groups in the polymer (i.e., the water molecules are nonfreezable). Under these circumstances, it can be difficult to observe freezable water via thermal techniques, and as a result, there are limited state of water data for low water content polymers. For this reason, the development of other techniques (e.g., NMR diffusometry^10^) that can contribute to characterizing the state of water in hydrated polymers would be helpful.

Dielectric relaxation spectroscopy (DRS) performed in the microwave frequency range is a technique that can be used to characterize the state of water in hydrated polymers.^13–17^ DRS in the microwave frequency range characterizes the relative permittivity properties of a material. These properties are related to a variety of physical phenomenon (e.g., ionic conductivity, dipolar (i.e., rotational) motion, and atomic/electronic polarization),^17,18^ and as a result, DRS can be used to inform molecular motions in hydrated polymers.

To probe water molecule dynamics (i.e., dipolar motions) in hydrated polymers, samples are subjected to an oscillating electromagnetic field in the microwave frequency range.^18^ In this frequency range, dipoles such as water molecules can polarize to store energy in the presence of an electromagnetic field (i.e., the dipoles orient in phase with the field). As the frequency of the field increases, the rate at which the field oscillates will, at some point, exceed the rate at which water molecules can reorient to stay in phase with the field, and this point is consistent with a so-called dipolar relaxation process where water molecules dis-orient with the field and the stored energy dissipates. Intra- and intermolecular interactions between water molecules and other moieties within the system can influence the time scale (i.e., observed frequency of occurrence) and extent of this relaxation process. These influences can be quantified to provide information about the state of water in a sample.

DRS in the microwave frequency range has been used previously to characterize the state of water in hydrated polymers such as Nafion^19,20^ (i.e., a commercially available perfluorinated sulfonated charge-containing polymer), charge containing sulfonated poly­(ether–ether ketones)^21^ and sulfonated poly­(arylene ether sulfones),^22,23^ and some model uncharged cross-linked polymers.^15,16^ A significant goal of these previous studies was to determine the low-frequency limit of the relative permittivity (i.e., the static dielectric constant) in these hydrated polymers, which is a material property that influences polymer ion transport properties.^17^ Within these studies, there are few examples where DRS data have been correlated to the state of water in hydrated samples in a manner analogous to techniques such as DSC, and as a result, there remain opportunities to understand how DRS can be further used to characterize the amounts of tightly bound, loosely bound, and bulk-like water in hydrated polymers.

Sulfonated poly­(arylene ether sulfones) are promising low-water content membrane materials for separation applications, and significant efforts have been made previously to characterize how structural modifications and processing techniques (e.g., variation of degree disulfonation and acidification of the sulfonate group) influence their ion and water transport properties.^7,10,22,24–30^ As discussed above, however, other techniques used to characterize the state of water are less suitable for low water content samples, and as a result, state of water data for these sulfonated polysulfones are limited to high water content samples (i.e., water volume fractions greater than 0.3).^10,11^ Here, we used microwave-frequency DRS to characterize the relative permittivity properties of two series of sulfonated poly­(arylene ether sulfones), BPS—XX, and BPSH—XX, over a wide range of water contents (i.e., water volume fractions ranging from 0.02 to 0.75) to probe how degree disulfonation and acidification influence the state of water in sulfonated polysulfones. Using these relative permittivity properties, we determined the hydration-dependent dielectric constant of the polymers and calculated the concentration of irrotationally bound (i.e., tightly bound), rotationally bound (i.e., loosely bound), and bulk-like water molecules in the sulfonated polysulfones. We also characterized the relative permittivity properties of hydrated Nafion, which we used as a comparison material for sulfonated polysulfones.

We found that generally the frequency-dependent relative permittivity properties of the polymers scaled inversely with the strength of the conjugate base in the hydrated polymers. In other words, we observed higher concentrations of restricted water molecules that could not contribute to dipolar relaxation processes and thus lower dielectric constants in polymers where the conjugate base was strongest; the relative ordering of tightly bound water concentration was BPS—XX > BPSH—XX > Nafion. Alternatively, the concentration of bulk-like water in the polymers followed the opposite ordering of Nafion > BPSH—XX > BPS—XX, which is also consistent with the physical picture where the strength of the conjugate base governs the state of water in the sulfonated polymers. These state of water results are generally consistent with observations made in sulfonated polymers using other state of water analysis techniques.^10,11^

Using a heterogeneous phase model-based analysis, we extracted information about the polymer morphology from the dielectric constant and water volume fraction data. This analysis suggested that a percolated water continuous network is present in Nafion, BPSH—XX, and BPS—XX at large water volume fractions. Furthermore, the critical water volume fraction onset of this percolation threshold is influenced by polymer chemistry (with a relative ordering of the critical water volume fraction of Nafion < BPSH—XX < BPS—XX), which is also consistent with previous observations.^28,31^

Finally, we characterized the salt transport (i.e., sorption, diffusion, and permeability) properties of the sulfonated polysulfones and used polymer dielectric constant data to describe how the thermodynamic environment in the sulfonated polymers contributes to polymer salt sorption properties. We found that as the polymer fixed charge concentration increased, the polymer dielectric constant decreased due to a dielectric saturation phenomenon. As a result, we hypothesized that the extent of dielectric exclusion in the polymers would increase with an increasing fixed charge concentration. The Donnan–Manning–Born model, which accounts for both dielectric and Donnan–Manning exclusion interactions,^25^ described the polymer salt partitioning data to a greater extent than the Donnan–Manning model alone, suggesting that modification of the polymer fixed charged concentration influences dielectric exclusion interactions.

Ultimately, these results advance the use of DRS to characterize the state of water in hydrated polymers. Our results and analysis suggest that modifications of polymer chemistry that influence the polymer charge density also influence polymer relative permittivity properties, and this variation in the electrostatic environment of the polymer influences polymer transport properties (e.g., dielectric and Donnan–Manning exclusion). These results and analysis unveil and explain the molecular underpinnings of interactions between polymer, ions, and water molecules in sulfonated polysulfones, which may be helpful to guide molecular engineering strategies for charged polymers as materials to control the rates of transport of ions and water molecules.

## 2 Materials and Methods

### 2.1 Materials

Poly­(arylene ether sulfone) random copolymers were synthesized using a nucleophilic step growth reaction reported previously.^29,32^ The polymers were synthesized to test the influence of two structural parameters on the relative permittivity properties of the hydrated polymers: (1) varied mole percent sulfonated comonomer relative to the nonsulfonated comonomers incorporated in the synthetic mixture (i.e., degree of disulfonation) and (2) precasting counterion form (i.e., acidification).

The mole percent sulfonated comonomer incorporated in the synthetic mixture was varied between 0% and 65% (Table 1). After the synthesis, the counterion form of the polymer was varied by subjecting the polymers to acidification. In this process, membranes cast from the polymers were acidified by boiling in 0.2 M sulfuric acid for 2 h and then were soaked in deionized water for 2 h. This process resulted in polymers where the fixed charge groups were in the protonated (i.e., acid) counterion form. In unacidified polymers, the as-cast polymers are predominantly in the potassium counterion form. The nomenclature used for the copolymers is BPS—XX or BPSH—XX, where BPS stands for biphenol-based poly­(arylene ether sulfone)­s, XX denotes the mole percent of sulfonated comonomers, and H is used to denote that the copolymers are in the acid form (Table 1). For example, BPSH—20 is a copolymer in the acid counterion form with 20 mol % of the sulfonated comonomer and 80 mol % of the nonsulfonated comonomer.

**Table 1:**
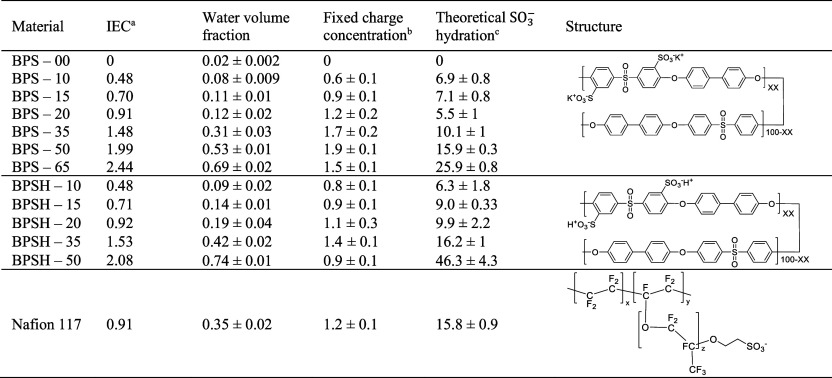
Material Properties of the BPS—XX, BPS—XX, and Nafion Polymers

a Units: meq (R–SO_3_^–^)/g (dry polymer).

b Units: eq (R–SO_3_^–^)/L (swollen polymer).

c Units: mol (water)/eq (R–SO_3_^–^).

The poly­(arylene ether sulfone) copolymers were cast into free-standing films from a 10 wt % solution of polymer in DMAc (i.e., [g (polymer)/g (DMAc)]). After dissolving the polymer, the solution was drop-cast onto a glass plate using a glass pipet or syringe. The film was then left under a heat lamp for 24–48 h to evaporate the solvent. Next, the film was placed into a vacuum oven at 110 °C for 24 h to facilitate removal of solvent. The membranes were then removed from the plate and either kept in the as-cast salt form or acidified, as described earlier.

The Nafion 117 films were pretreated according to previously reported procedures.^33^ First, the polymer was soaked in 3 wt % H_2_O_2_ (ACS reagent, Thermo Scientific) for 1 h at 80 °C, after which the polymer was soaked in aqueous 0.1 M H_2_SO_4_ (ACS reagent, Sigma-Aldrich) for 1 h at 80 °C. Finally, the membrane was soaked in deionized (DI) water for 1.5 h at 80 °C, and over this time, the water was replaced with fresh DI water at least three times.

### 2.2 Dielectric Relaxation Spectroscopy (DRS)

Microwave dielectric relaxation spectroscopy (DRS) was used to characterize the frequency-dependent relative permittivity of the sulfonated polysulfones. This DRS measurement was performed as previously reported and is briefly summarized here.^13,17,25^ Hydrated polymers were subjected to an oscillating electromagnetic field in the microwave frequency range (45 MHz to 26.5 GHz), which was generated by a Keysight N9928A vector network analyzer (VNA). A 5 cm long, 3.5 mm diameter coaxial transmission line was used as the sample holder, and this line was connected to the VNA by using shielded coaxial cables. The VNA collected signals of the electromagnetic radiation reflected from and transmitted through the polymer samples. These signals were expressed as four scattering parameters, which were related to the relative complex permittivity of the samples as described previously.^13^

The geometry of the sample holder in the measurement requires that the hydrated polymer samples fill the annular space in the coaxial sample holder. To fill this annulus, hydrated polymer films were cut into 0.5 cm strips, blotted dry to remove excess surface water, and quickly wrapped tightly around the inner conductor until enough polymer was wrapped to completely fill the annular space of the sample holder. Care was taken to ensure that surface water droplets were completely removed and that there were no air gaps in the annular space of the sample holder to minimize the presence of excess water or air molecules in the sample holder during the measurement. Either surface water droplets or atmospheric air gaps can introduce experimental artifacts in the relative permittivity spectra,^13^ and after wrapping each sample around the inner conductor of the sample holder, the filled sample holder was carefully inspected to verify that there were no visible gaps between the polymer film and the sample holder.

### 2.3 Water Uptake and Dry Density

The water uptake and dry density of the hydrated polymers were determined via a hydrostatic weighing technique reported previously.^13^ The BPS XX and BPSH XX materials were equilibrated in deionized water or 1 M NaCl for at least 72 h. These equilibration solutions were changed at least three times to ensure that the equilibration procedure proceeded to its full extent. The samples were then removed from solution, dried of surface water droplets, and quickly weighed to determine their hydrated mass, *m*_wet_. These hydrated samples were dried under vacuum at 80 °C for at least 48 h. After this drying procedure, the samples were weighed to obtain their dry mass, *m*_dry_, and the sample water uptake, *w*_u_, was determined as^13^

1 $$w_{u}=\frac{m_{wet}-m_{dry}}{m_{dry}}$$

To determine the dry density of the samples, the dry polymers were submerged and weighed in cyclohexane, which is an auxiliary solvent that does not readily partition into a sulfonated polysulfone. Using this auxiliary mass, *m*_aux_, the dry density was calculated from Archimedes’ Principle as^13^

2 $$\rho_{p}=\frac{m_{aux}}{m_{aux}-m_{air}}\left(\rho_{aux}-\rho_{air}\right)$$

where ρ_aux_ and ρ_air_ are the dry densities of cyclohexane and air, respectively. Finally, the volume fraction of water in the polymer, φ_w_, was calculated using a volume additivity assumption^13^

3 $$\varphi_{w}=\frac{w_{u}}{w_{u}+\rho_{w}/\rho_{p}}$$

where ρ_w_ is the density of water. This water volume fraction is related to the concentration of water in the polymer at equilibrium, $c_{w}^{m}$ , in units of [mol (water)/L (swollen polymer)] as

4 $$c_{w}^{m}=\frac{\varphi_{w}\rho_{w}}{M_{w}}$$

where *M*_w_ is the molecular weight of water.

### 2.4 Salt Transport Properties

The NaCl permeability coefficient of the polymer samples was directly measured using a previously reported direct salt transport method.^24^ First, the thickness of the hydrated polymer samples was measured using a pair of digital calipers (Item No. 293-344, Mitutoyo). Then, the hydrated polymer samples were clamped between two jacketed 32 mL permeation cells with a 1.5 cm^2^ active area for transport (Side-Bi-Side Cells, Permegear, Hellertown, PA, USA). The cells initially contained either 1 M NaCl (i.e., the donor cell) or deionized water (i.e., the receiver cell) and were kept at a constant temperature of 25 ± 0.1 °C. These cells were continuously stirred using magnetic stir bars to homogenize each solution.

The concentration of salt in the receiver cell was determined by using a conductivity probe (LR325/01, WTW, Germany) to monitor the conductivity of the solution in the receiver cell, which was then converted to the concentration of NaCl in the solution by using a calibration curve that was valid at the experimental temperature. The time-dependent concentration of the receiver cell, *C*_R_(*t*), was used to determine the salt permeability coefficient, *P*_s_, via^24^

5 $$\ln\left[1-2\frac{C_{R}\left(t\right)}{C_{D}\left(0\right)}\right]\left[-\frac{Vl}{2A_{s}}\right]=P_{s}t$$

where *t* is the experimental time, *C*_D_(0) is the initial concentration of the donor cell at time (which is approximately equal to its value over the course of the experiment), *A*_s_ is the active area of the cell (1.5 cm^2^), *V* is the cell volume, and *l* is the polymer thickness. Equation 5 is valid in the pseudosteady state limit of the experiment.^24^

The salt sorption properties of the polymers were subsequently measured by using a commonly reported desorption technique. The polymer samples were first equilibrated in 1 M NaCl for at least 72 h, and over this time scale, these equilibration solutions were replaced at least three times to ensure that the polymer was fully equilibrated with the external solution. The polymer thickness and diameter were then quickly measured after removal from the equilibration solution. Finally, polymers were blotted dry to remove excess NaCl solution droplets from the polymer surface and placed in 20 mL of deionized water for 24 h, where NaCl desorbs from the polymer into the solution. The temperature of the desorption solution and polymer was kept constant at 25 °C by placing the solutions in a temperature-controlled chamber. The time scale of the experiment was chosen to be in excess of an estimated characteristic time scale for NaCl diffusion in the polymers, which was determined using the previously measured permeability coefficients as a proxy for the diffusion coefficient. Generally, the permeability coefficients in sulfonated polysulfone are approximately an order of magnitude smaller than their diffusion coefficients,^24^ so this estimated diffusion time scale is expected to be sufficiently large to allow the desorption process to proceed to its full extent.

After 24 h, the concentration of the desorption solutions was measured using the previously described conductivity probe and calibration curve. The concentration of the desorption solution was then related to the salt sorption coefficient of the polymer, *K*_s_, as^13,24^

6 $$K_{s}\equiv \frac{C_{s}^{m}}{C_{s}^{s}}=\frac{C_{s}^{d}V_{d}}{C_{s}^{s}V_{p}}$$

where *C*_s_^m^ is the equilibrium concentration of salt in the membrane after its initial equilibration but before desorption, *C*_s_^s^ is the concentration of salt in the solution that the polymer is initially equilibrated with (i.e., 1 M NaCl), *C*_s_^d^ is the final concentration of salt in the desorption solution, and *V*_d_ and *V*_p_ are the volumes of the desorption solution and polymer, respectively. The units of the salt sorption coefficient in eq 6 are [(mol/L (swollen polymer))/(mol/L (solution))], which are relevant for the analysis of flux-dependent properties that require knowledge of the total polymer volume (e.g., permeability).

## 3 Results and Discussion

### 3.1 Relative Permittivity Properties and State of Water

As discussed in the introduction, the molecular environment of a water molecule influences its ability to orient with (i.e., polarize in the presence of) an oscillating electromagnetic field in the microwave frequency range.^18^ For example, in hydrated polymers, the dipolar (i.e., rotational) motions of water molecules that experience strong attractive interactions with hydrophilic moieties on the polymer backbone (e.g., charged sulfonate groups) are more restricted than bulk-like water that is free to rotate in the presence of an electromagnetic field.^17,19^ As a result, bound water molecules in a hydrated polymer relax at lower frequencies (i.e., have slower rotational motions) and are generally less polarizable than bulk-like water molecules in a hydrated polymer. In hydrated Nafion, two distinct dipolar relaxation processes, at approximately 1 and 20 GHz, are observed in DRS measurements (Figure 1), suggesting that, based on the previously described physical picture, distinct populations of bound and bulk-like water molecules are present in hydrated Nafion (Figure 1). Two distinct dipolar relaxation processes are also observed in DRS measurements of the sulfonated polysulfones, suggesting that there are at least two distinct water molecule populations in the BPS—XX and BPSH—XX polymers as well (Figure 2A,B).

**Figure 1:**
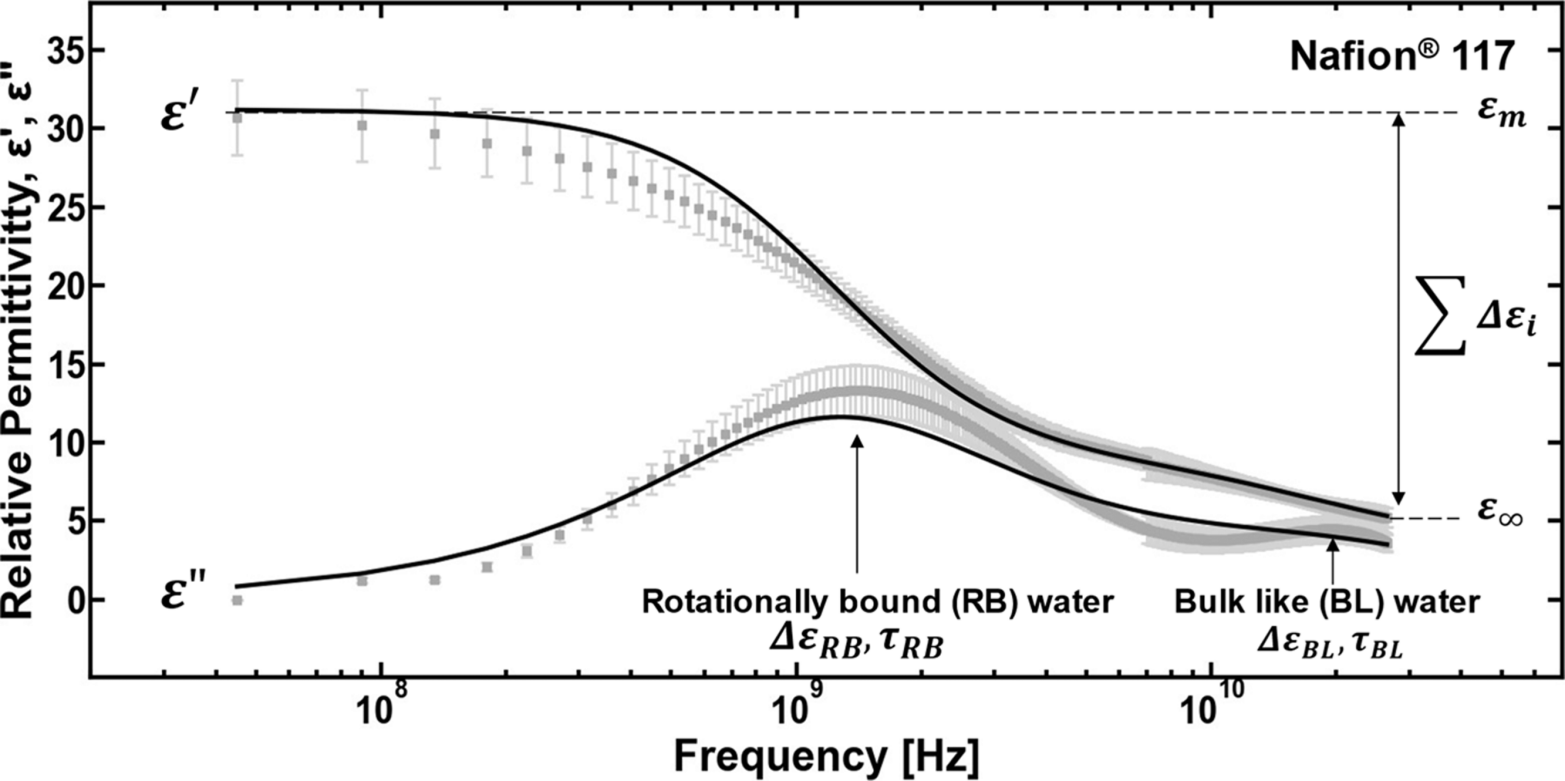
Frequency-dependent real and imaginary parts of the complex relative permittivity of hydrated Nafion 117. The solid lines are plotted as the summation of two Debye relaxation processes that were simultaneously fit to the real and imaginary parts of the complex relative permittivity spectra. All measurements were made at room temperature (i.e., 25 ± 1 °C), and the uncertainty was taken as the standard deviation from the mean of three measurements.

**Figure 2:**
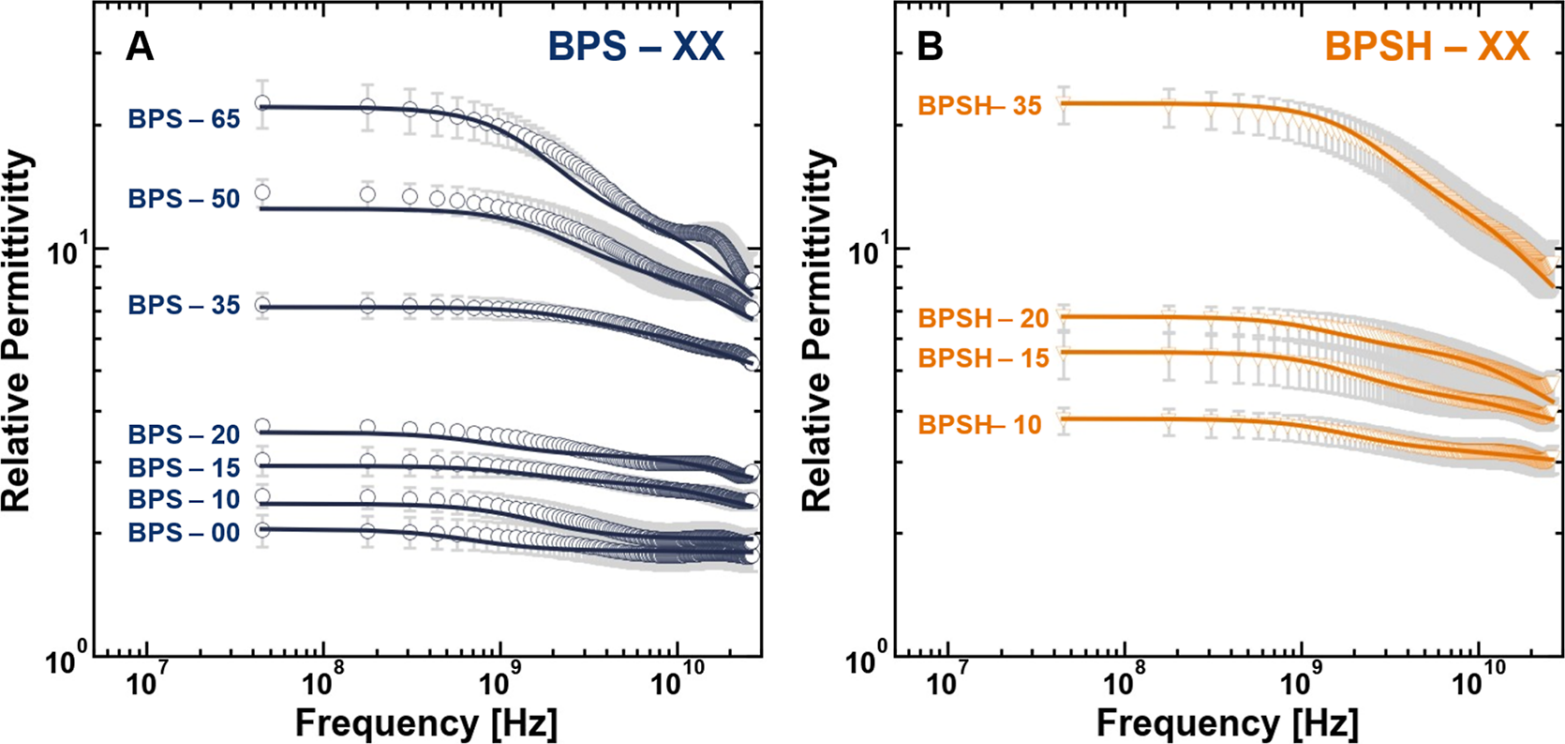
Frequency-dependent real part of the complex relative permittivity of (A) hydrated BPS—XX and (B) BPSH—XX. The solid lines are plotted as the summation of two Debye relaxation processes that were simultaneously fit to the real and imaginary parts of the complex relative permittivity spectra. All measurements were made at room temperature (i.e., 25 ± 1 °C), and the uncertainty was taken as the standard deviation from the mean of three measurements.

The frequency-dependent relative permittivity can be used to quantify the extent to which polymer–water interactions perturb dipolar relaxation processes in hydrated polymers. For example, the characteristic relaxation time, τ_i_, of the dipolar relaxation processes describes rotational water molecule dynamics when subject to the electromagnetic field, and this time scale for water molecules in the hydrated polymer can be compared to that in the bulk to characterize how interactions with the polymer influence the molecular motions of water molecules. This characteristic time is related to the frequency where the dipolar relaxation is observed in a DRS measurement (i.e., the frequency of the peak in the imaginary part of the complex relative permittivity), as τ_j_ = 1/(2π*f*_maxloss_) (Figure 1).^17^ Likewise, the dielectric strength of a dipolar relaxation, Δε_*j*_, is related to the number of water molecules that participate in the dipolar relaxation process (Figure 1). This strength corresponds to the height of the loss peak (Figure 1) and can be used to extract information about the number of bound or bulk-like water molecules in the polymer matrix.^17^

These parameters are commonly determined by fitting the frequency-dependent relative permittivity data to a dipolar relaxation model.^18^ Here, we fit the frequency-dependent complex permittivity, ε*, to a summation of two Havriliak–Negami relaxation processes^19^

7 $$\varepsilon^{*}=\varepsilon_{\infty }+\sum_{j=1}^{2}\frac{\Delta \varepsilon_{j}}{{\left[1+{\left(i\omega \tau_{j}\right)}^{1-\alpha_{j}}\right]}^{\beta_{j}}}$$

In this relaxation model, *j* is an arbitrary summation variable representing the *j*th relaxation process, ε_∞_ is the high-frequency static permittivity, ω is the angular frequency, and α_*j*_ and β_*j*_ are empirically determined shape parameters. The best fit for all data were obtained with the shape parameters α_*j*_ = 0 and β_*j*_ = 1, which represents a special case of the Havriliak–Negami model where the DRS data are described by ideal Debye relaxation processes.^18^ The choice to constrain the model to two relaxation processes—a nonrestricted (i.e., a bulk-like) relaxation where τ = 8.3 × 10^–12^ seconds and a restricted relaxation where τ > 8.3 × 10^–12^—was effectively made to reduce the number of fitted parameters in the model while still capturing the general physics associated with the dipolar relaxation process.^19^ For example, while it is possible that more than two states of water with distinct relaxation dynamics exist in these hydrated polymers (indeed, this claim may be supported by the observation that the two-state Havriliak–Negami fits do not describe perfectly all the relative permittivity data for the sulfonated polysulfones and Nafion (Figures 1 and 2)), accounting for these states of water would significantly increase the degrees of freedom in the model, which may not describe the experimental data in a meaningful way.

Physical phenomenon other than dipolar relaxations, such as ionic conduction, can map on to the relative permittivity spectra of hydrated polymers as well, and these phenomena can obscure dipolar motions in the low-frequency limit of the measurement.^18^ To minimize the influence of ionic conduction on the data analysis, which is significant in these charge-containing polymers,^20,21^ we used a Kramers–Kronig analysis procedure to approximate the conductivity-free imaginary component of the relative permittivity as^18^

8 $$\varepsilon^{\prime \prime }\approx -\frac{\pi }{2}\frac{\partial \varepsilon^{\prime }\left(\omega \right)}{\partial \ln\omega }$$

This Kramers–Kronig analysis is advantageous because it allows data to be fit to the Havriliak–Negami model using fewer fit parameters than other techniques used to remove artifacts of ionic conduction from the permittivity spectra (e.g., using a fitted conductivity term).^20^

For convenience, the parameters determined from the fitting procedure are listed in Table 2. Subsequently, we use these parameters to extract information about the state of water and dielectric constant in the sulfonated polysulfones and Nafion. These parameters can also be used to determine the dielectric constant, which is an important material property useful for modeling ion transport in hydrated polymers.

**Table 2:** Parameters Determined by Fitting the DRS Data for BPS—XX, BPSH—XX, or Nafion to the Havriliak–Negami Relaxation Model (Equation 7)

material	**ε**_**∞**_	Δ**ε**_**BL**_	Δ**ε**_**RB**_	**τ**_**BL**_	**τ**_**RB**_
				[picoseconds]	[picoseconds]
BPS—00	1.8 ± 0.1	0.02 ± 0.01	0.23 ± 0.02	8.3	240 ± 11
BPS—10	1.9 ± 0.2	0.01 ± 0.01	0.42 ± 0.01	8.3	100 ± 25
BPS—15	2.2 ± 0.1	0.51 ± 0.02	0.26 ± 0.02	8.3	110 ± 24
BPS—20	2.6 ± 0.1	0.56 ± 0.04	0.43 ± 0.04	8.3	182 ± 14
BPS—35	4.7 ± 0.1	1.46 ± 0.10	0.99 ± 0.10	8.3	51 ± 32
BPS—50	5.6 ± 0.1	2.98 ± 0.22	3.8 ± 0.2	8.3	70 ± 21
BPS—65	5.6 ± 0.3	5.81 ± 0.54	10.6 ± 0.5	8.3	92 ± 28
BPSH—10	2.9 ± 0.1	0.26 ± 0.02	0.62 ± 0.01	8.3	89 ± 19
BPSH—15	3.5 ± 0.1	0.82 ± 0.03	1.21 ± 0.02	8.3	84 ± 14
BPSH—20	3.5 ± 0.1	2.17 ± 0.03	1.16 ± 0.03	8.3	104 ± 11
BPSH—35	5.6 ± 0.3	6.79 ± 0.20	10.3 ± 0.31	8.3	57 ± 6
Nafion 117	3.5 ± 0.1	5.16 ± 0.14	22.6 ± 0.14	8.3	123 ± 2.0

#### 3.1.1 State of Water

Sulfonated polysulfones and Nafion are composed of hydrophobic backbones (e.g., aryl-ether or Teflon backbones, respectively) decorated with hydrophilic side chains and/or sulfonate groups. In these polymers, it is reasonable to assume that water/polymer interactions are governed primarily by interactions between water molecules and the sulfonate groups, and under these circumstances, the state of water can be described using a model consisting of three water molecule populations: bulk-like (BL) water, rotationally bound (RB) water, and irrotationally bound (IB) water (where RB and IB water molecules are assumed to be bound to sulfonate groups) (Figure 3). Each of these populations interacts with the sulfonate groups to different extents, and these interactions influence the ability of water molecules to polarize during the DRS measurement (Figure 3). These three states generally correspond to free water molecules, loosely bound water molecules, and tightly bound water molecules (i.e., headgroup waters), respectively, described elsewhere.^10–12^

**Figure 3:**
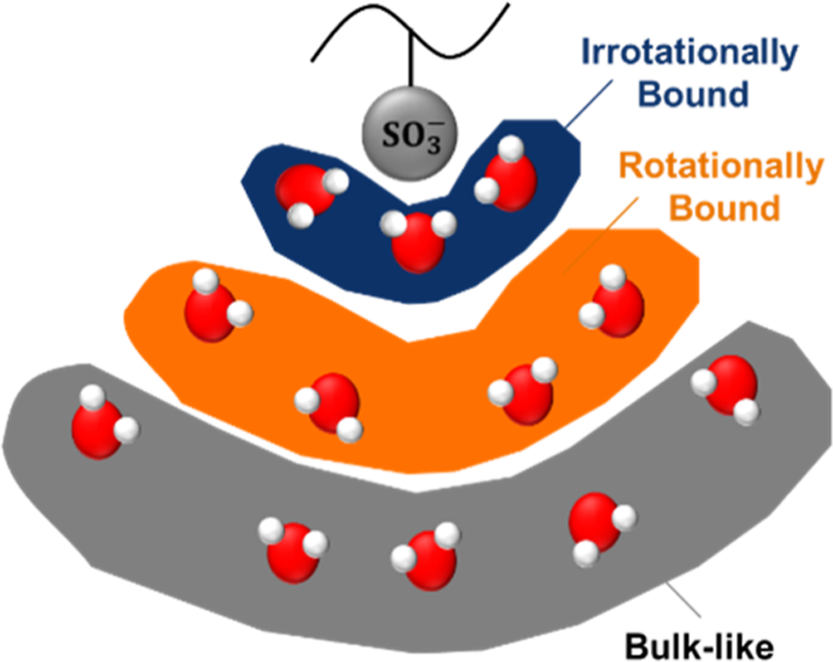
Cartoon representing the bulk-like, rotationally bound, and irrotationally bound water molecules in the sulfonated polysulfones. The number of water molecules and orientation shown in the schematic is not necessarily representative of the actual water molecule populations. Adapted from Solid State Ionics, 275, Smedley et al., Measuring water hydrogen bonding distributions in proton exchange membranes using linear Fourier Transform Infrared spectroscopy, 66–70, Copyright 2015, with permission from Elsevier.^12^

As discussed previously, the different states of water can be distinguished from each other in the DRS measurement based on their characteristic relaxation time. The dipolar relaxation process observed at 20 GHz is attributed to bulk-like water in the polymer (i.e., τ_BL_ = 8.3 ps) because in bulk solutions subject to DRS, hydrogen-bonded water disorients at a frequency of approximately 18–20 GHz (Figure 1).^19,34^ A statistically significant relaxation process with a characteristic relaxation time of 8.3 ps was observed for most of the polymers (Table 2), suggesting that there was bulk-like water present in most materials.

The dipolar relaxation process observed at frequencies less than 20 GHz is attributed to rotationally bound water molecules (i.e., τ_RB_ > 8.3 ps) (Figure 1). The physical picture for these water molecules is that they interact (i.e., solvate) the sulfonate groups to an extent that restrict their rotational motions and cause them to disorient at lower frequencies than bulk-like water but that the strength of this interaction does not prevent these water molecules from polarizing in the presence of the electromagnetic field (Figure 3). This second relaxation process, with a characteristic relaxation time larger than that of the bulk, was observed for each polymer as well, suggesting that there is also a significant amount of rotationally bound water molecules in each of the sulfonated polysulfones (Table 2). Generally, we observed no systematic trends underscoring the intermaterial differences in these relaxation times (Table 2), and as a result, we were unable to determine how polymer backbone dynamics (i.e., Nafion compared with the sulfonated polysulfone) or structural modifications of the sulfonated polysulfones influenced the dynamics of the rotationally bound water molecule process.

Finally, under circumstances where the rotational motions of water molecules are sufficiently restricted by intra- or intermolecular interactions that they can no longer polarize in the presence of an electromagnetic field, they are considered irrotationally bound. Water molecules with these properties are observed in electrolyte solutions, ice, and may also be present in the charged polymers considered here.^18,35^ While, by nature, these water molecules are invisible to DRS, their relative concentration in a sample can be determined given the apparent concentrations of the other water molecule populations in the sample as

9 $$c_{w}^{m}=c_{w,BL}^{m}+c_{w,RB}^{m}+c_{w,IB}^{m}$$

where *c*_w,*j*_^m^ is the concentration of water in state *j*.

The concentration of bulk-like or rotationally bound water molecules can be determined from the frequency-dependent relative permittivity data using a Kirkwood–Froelich equation-based approach reported by Buchner et al.^35^

10 $$c_{w,j}^{m,ap}=c_{w}^{s}F_{KF}\frac{2\varepsilon_{m}+\varepsilon_{\infty }}{\varepsilon_{m}}\Delta \varepsilon_{j}$$

where ε_m_ is the low-frequency limit of the relative permittivity (i.e., the static dielectric constant, which is discussed in greater detail in Section 3.1.3) and *c*_w_^s^ is the concentration of water in the external solution (i.e., 1 g/cm^3^ (solution)). The superscript ap is used to denote that eq 10 is calculated by normalizing the Kirkwood–Froehlich equation written for a specific relaxation process to that of deionized water, which is useful because it minimizes ambiguities in the choice of the cavity and reaction fields used to calculate the Kirkwood correlation factor for water sorbed in the polymer matrix, and provides a common basis for intermaterial analysis.^35^ The function *F*_KF_ is related to the relative permittivity properties of deionized water as^35^

11 $$F_{KF}=\frac{\varepsilon_{s}}{\left(\varepsilon_{s}-\varepsilon_{\infty }^{s}\right)\left(2\varepsilon_{s}+\varepsilon_{\infty }^{s}\right)}$$

where ε_s_ and ε_∞_^s^ are the low- and high-frequency limits of the relative permittivity of deionized water.

To fully describe each state of water in the polymers (i.e., calculate *c*_w,IB_^m^ from *c*_w,BL_^m^ and *c*_w,RB_^m^) (eq 9), the total concentration of water in the hydrated polymers was first calculated from the polymer water volume fraction (eq 4). In sulfonated polymers, the equilibrium water content can be influenced by factors such as the degree of disulfonation and counterion form of the fixed charge group and the pretreatment used to prepare the polymers (i.e., the acidification process).^10,24,36^ Generally, as the degree of sulfonation increases (i.e., the ion exchange capacity increases), the equilibrium water content of the polymers increases, as well. This result also is expected based on previous observations in the literature, where the water content increases with increasing ion exchange capacity, and these results are consistent with a physical picture where the equilibrium concentration of water in the polymer effectively increases with ion exchange capacity to fully solvate the increasing number of fixed charges in the polymer matrix.

The concentrations of irrotationally bound, rotationally bound, and bulk-like water also increase with increasing ion exchange capacity (Figure 5). Observations that the concentrations of the bound water molecule populations increase with increasing ion exchange capacity support the physical picture that generally, the increased water sorption of the hydrated polymers with increasing IEC facilitates solvation of the hydrophilic sulfonate groups in the polymer matrix. Observations that the concentration of bulk-like water molecules is nonzero for all polymers suggest that water molecules partition into the polymer matrix in excess of what is required to solvate the sulfonate groups (Figure 5).

At a given ion exchange capacity, the relative ordering of the total water concentration in the polymers is Nafion > BPSH—XX > BPS—XX (Figure 4). This relative order is maintained in the concentrations of rotationally bound and bulk-like water concentrations (Figure 5B,C). However, it changes in the concentration of irrotationally bound water, which was generally identical for BPS—XX and BPSH—XX at most IECs and zero for Nafion (Figure 5A). These results are generally consistent with previous observations that water is generally more bulk-like (i.e., less irrotationally bound) in Nafion than in sulfonated poly­(aryl ether sulfones).^10^

**Figure 4:**
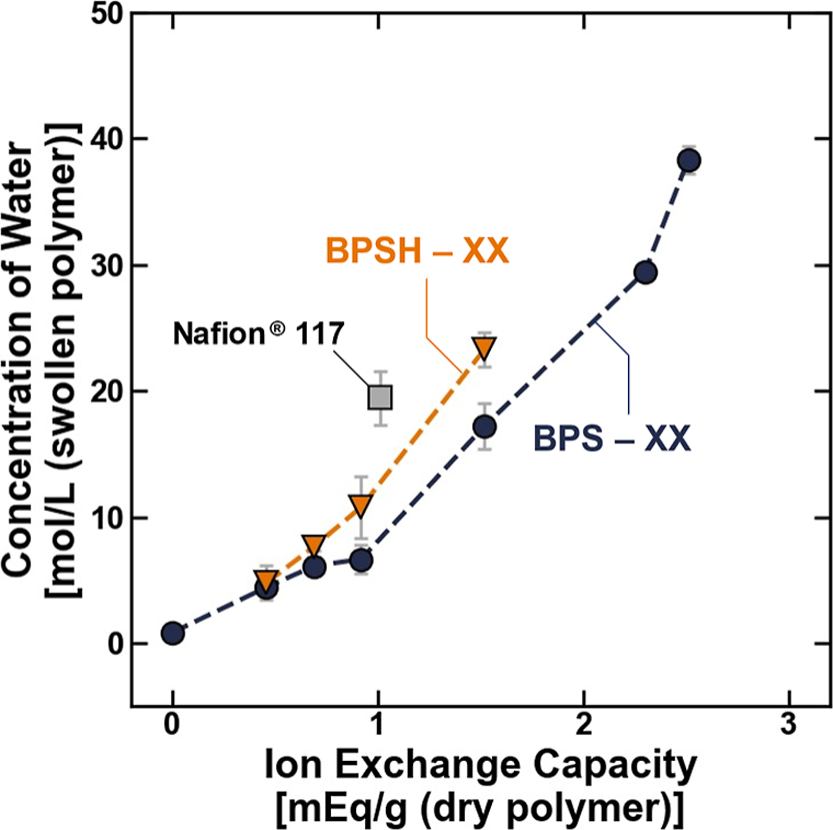
Total concentration of water in the BPS—XX (blue ●) and BPSH—XX (orange ▼) polymers plotted as a function of the ion exchange capacity. The lines are drawn to guide the eye. All measurements were made at room temperature (i.e., 25 ± 1 °C), and the uncertainty was taken as the standard deviation from the mean of three measurements.

**Figure 5:**
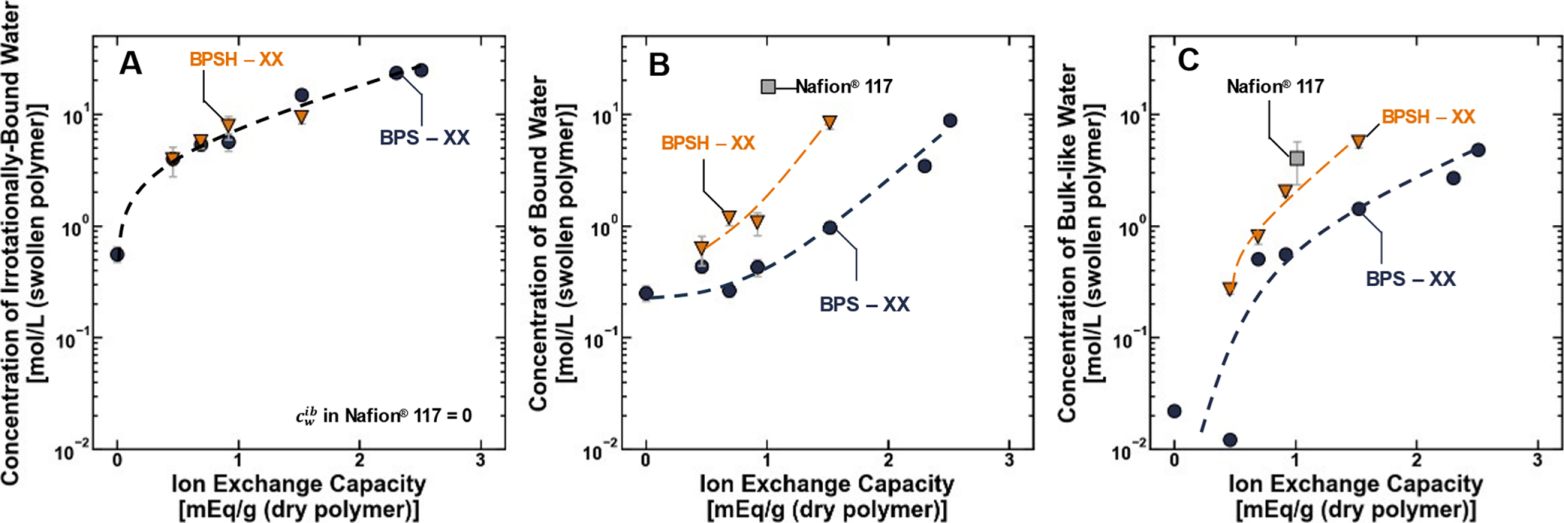
Concentrations of (A) irrotationally bound, (B) rotationally bound, and (C) bulk-like water in the BPS—XX (blue ●) and BPSH—XX (orange ▼) polymers plotted as a function of the polymer ion exchange capacity. The lines are drawn to guide the eye. All measurements were made at room temperature (i.e., 25 ± 1 °C), and the uncertainty was propagated from the relative permittivity and water uptake measurements.

These results may be partially explained using a physical picture based on previous observations where the peroxide-based pretreatment of Nafion and acidification via boiling in sulfuric acid of the polysulfones expand the hydrophilic domains in the sulfonated polymers to increase their water content.^10,11,37,38^ Within this context, the relative ordering of the size of these hydrated domains at a given ion exchange capacity, which are generally on the order of 1–10 nm in size, is generally Nafion > BPSH—XX > BPS—XX.^28,37,38^ Under these circumstances, the relative ordering of the total and bulk-like water concentrations appears to be consistent with a physical picture where the total water content increases as the size of the hydrophilic domain increases and water molecules may be, on average, located further away from the hydrophilic sulfonate groups that impede their molecular motions.

However, this physical picture does not fully explain the observed differences in the concentrations of irrotationally and rotationally bound water molecules. The concentration of bound water, which is likely closer to the sulfonate groups than the bulk-like water in the hydrophilic domain, may be expected to be influenced less significantly by morphological factors than by water/charge interactions that occur close to the fixed charges (i.e., within the length scale of a water molecule, which is approximately 0.1 nm).^8^ As a result, the observed relationship between the polymer structure and the concentration of the bound water molecules in the polymer may be explained by using an analysis of how chemistry-based factors (e.g., the strength of the conjugate base of the sulfonic moieties) may influence the water/fixed charge interactions.

The concentration of irrotationally bound water molecules is inversely proportional to the strength of the conjugate base in the charged polymers (i.e., the relative ordering is BPS—XX ∼ BPSH—XX > Nafion) (Figure 5A). In Nafion, the sulfonate group is tethered to the polymer via a super acid perfluorinated side chain, which results in a very strong sulfonic acid and weaker conjugate base.^10^ In other words, the effective charge density of the sulfonate group in Nafion is reduced, resulting in weaker hydrophilic interactions with the surrounding water molecules (and no irrotationally bound water; Figure 5A). Alternatively, in BPSH—XX, the sulfonic acid is tethered directly to the polymer backbone, which results in a stronger conjugate base (i.e., a higher negative charge density of the sulfonate group),^10^ and under these circumstances, the densely charged sulfonate group of BPSH—XX has stronger hydrophilic interactions with the surrounding water molecules and a larger concentration of irrotationally bound water molecules relative to Nafion (Figure 5A). To a first approximation, the sodium form sulfonate group in BPS—XX may be approximated as a strong conjugate base relative to the acid-form sulfonate groups in BPSH—XX and Nafion (i.e., the sodium-form sulfonate is more stable than the acid-form sulfonate, and thus, the sulfonate groups in hydrated BPS—XX are likely worse proton acceptors relative to those in hydrated BPSH—XX). For most of the BPS—XX and BPSH—XX polymers, at low ion exchange capacity, the irrotationally bound water concentrations of the BPS—XX and BPSH—XX polymers are statistically identical; however, at the largest comparable ion exchange capacity, the concentration of irrotationally bound water in BPS—XX is larger than that of BPSH—XX, which agrees with the proposed trend that the concentration of irrotationally bound water molecules scales directly with the strength of the conjugate base.

The concentration of bulk-like water in the polymers scales inversely with the strength of the conjugate base (i.e., is largest in Nafion and smallest in BPS—XX) (Figure 5C), and this result can also be explained using the analysis of the strength of the conjugate base (i.e., there is more bulk-like water in polymers with weakly charged sulfonate groups). The concentration of rotationally bound water molecules follows the same ordering as that of the bulk-like water molecules. This result is interesting because, as outlined in the preceding paragraph, one may expect the concentration of bound waters to scale inversely with the strength of the conjugate base. These observations may suggest that in polymers with a weaker conjugate base (i.e., Nafion relative to the sulfonated polysulfones), the lack of tightly, irrotationally bound water molecules in the sulfonate group’s hydration shell is supplemented by loosely, rotationally bound water molecules.

Comparisons of the relative amounts of irrotationally bound, rotationally bound, and bulk-like water molecules (i.e., the concentrations determined via DRS normalized by the total water molecule concentration determined via the equilibrium swelling experiments) are useful to describe the general state of water in the sulfonated polymers (Figure 6). This analysis suggests that most water molecules in the sulfonated polysulfones are irrotationally bound (i.e., the fraction of irrotationally bound water is greater than 0.5 in most samples) (Figure 6). The concentration of rotationally bound water is generally the second largest population in the sulfonated polysulfones, and the bulk-like water is generally the smallest population (Figure 6). Alternatively, for Nafion (where none of the water molecules are irrotationally bound), the rotationally bound water is the largest population, followed by bulk-like water (Figure 6). These results are consistent with the previous explanations based on the strength of the conjugate base in the polymers.

**Figure 6:**
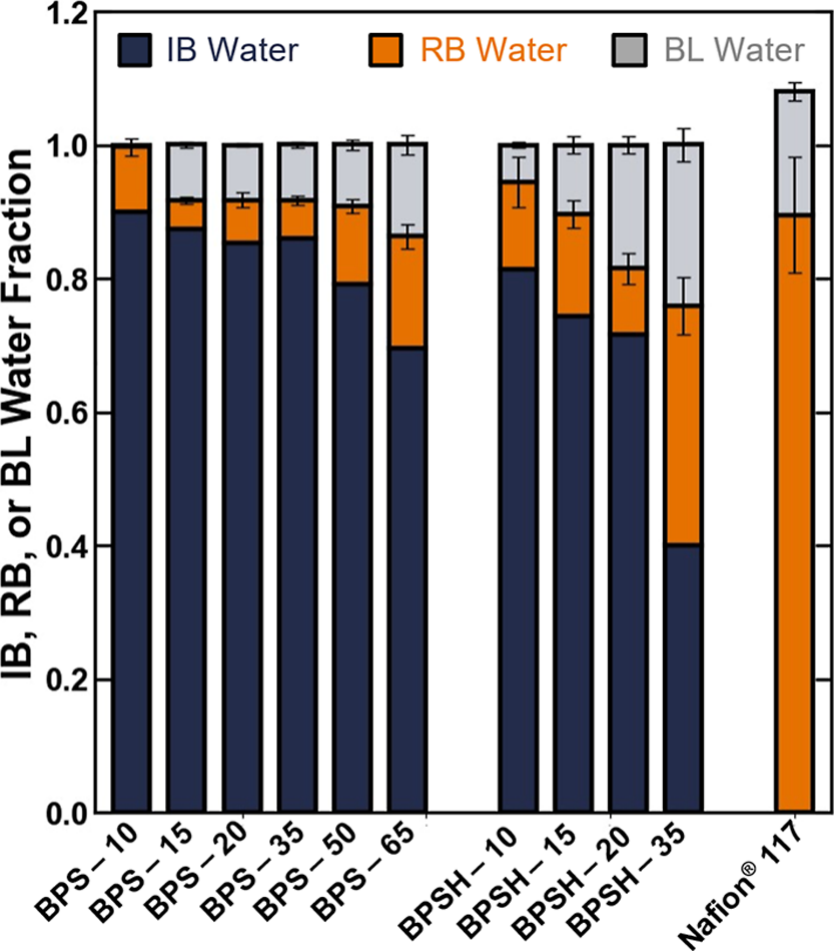
Concentration of irrotationally bound (blue bars), rotationally bound (orange bars), and bulk-like water (gray bars) in the BPS—XX, BPSH—XX, and Nafion polymers relative to the total concentration of water in the polymer.

It is noteworthy that the total relative concentration of water in Nafion exceeds unity (i.e., the sum of all the bars for Nafion in Figure 6 is greater than 1). This observation effectively suggests that the average concentration of water in Nafion determined from the DRS measurement using eq 9 is larger than the average concentration of water in Nafion determined via the equilibrium swelling experiments. This observed discrepancy between the data is only apparent, however, because within the experimental error, which was propagated from both the DRS and swelling measurements, the relative concentration is not statistically different from unity.

As discussed previously, several studies have characterized the states of water in sulfonated polysulfones and Nafion by using thermal techniques (e.g., DSC and TGA) as well as other techniques (e.g., NMR). Observations made in this state of water analysis via DRS, where bulk-like water generally makes up the smallest fraction of the total water sorbed in the polymer, are consistent with previous measurements using thermal techniques.^10,11^ However, the relative ordering of loosely bound and tightly bound water (or water that froze below 0 °C and water that did not freeze at all, respectively) is generally different relative to what is measured here for rotationally and irrotationally bound water measured via DRS (i.e., the concentration of loosely bound water is generally larger than the concentration of tightly bound water in DSC, but the concentration of irrotationally bound water is generally larger than the concentration of rotationally bound water in DRS).^10,11^

These results may be consistent with a physical picture where, based on the time scale of the experiment used to characterize the state of water, different populations of water molecules appear “tightly” or “loosely” bound in different measurements. For example, the irrotationally bound water molecules in DRS are likely not fully immobilized despite being nonpolarizable because water molecules present in solvation shells of charges can exchange with bulk-like water in the polymer matrix.^39^ If these exchange dynamics are slower than the time scale of the measurements, these water molecules would be invisible to DRS and thus contribute to the irrotationally bound water molecule populations. Alternatively, other techniques (e.g., thermal techniques) may probe water molecule dynamics over different time scales relative to DRS, and as a result, different techniques may allow for more water molecules to sample more molecular environments over the length scale of the experiment. Future investigations further probing how different techniques used to characterize the state of water in hydrated polymers (e.g., DSC and DRS) complement each other may be useful to specify the state of water in hydrated polymers.

#### 3.1.2 Sulfonate Group Hydration

A considerable advantage of DRS measurements is that DRS data can be used to directly characterize the number of solvent molecules that hydrate a solute (i.e., can be used to directly measure hydration numbers).^35^ In the absence of a direct measurement, the theoretical hydration number of a sulfonate group in a charged polymer, $\lambda_{SO_{3}^{-}}^{\max}$ , is calculated from the water content as $\lambda_{SO_{3}^{-}}^{\max}=c_{w}^{m}/c_{SO_{3}^{-}}^{m}$ . Using the concentrations of rotationally and irrotationally bound water molecules, and by assuming that the molecular motions of these water molecules are restricted due to interactions with sulfonate groups (and not other hydrophilic functionalities along the polymer backbone), the total DRS hydration number for the sulfonate groups in the polymer, $\lambda_{SO_{3}^{-}}^{DRS}$ can be calculated as

12 $$\lambda_{SO_{3}^{-}}^{DRS}=\frac{\left(c_{w,RB}^{m,ap}+c_{w,IB}^{m,ap}\right)}{c_{SO_{3}^{-}}^{m}}$$

The theoretical sulfonate group hydration generally increases with water content (i.e., increases with the degree of disulfonation and acidification) (Table 1). The experimentally determined total DRS sulfonate group hydration increases with degree disulfonation and acidification as well and, as a result, scales with the theoretical hydration number (Figure 7A). For most materials, the total DRS hydration number is approximately equal to the theoretical hydration number (i.e., most data points fall on the parity line in Figure 7A), which may be explained by the observation that bulk-like water is generally the smallest water molecule population for the sulfonated polymers (Figure 6). These results are consistent with a physical picture, where the dynamics of most of the water molecules in the sulfonated polysulfones are perturbed by interactions with sulfonate groups. The total DRS sulfonate group hydration number was not influenced by polymer chemistry (i.e., BPS—XX, BPSH—XX, and Nafion generally fall on the same line).

**Figure 7:**
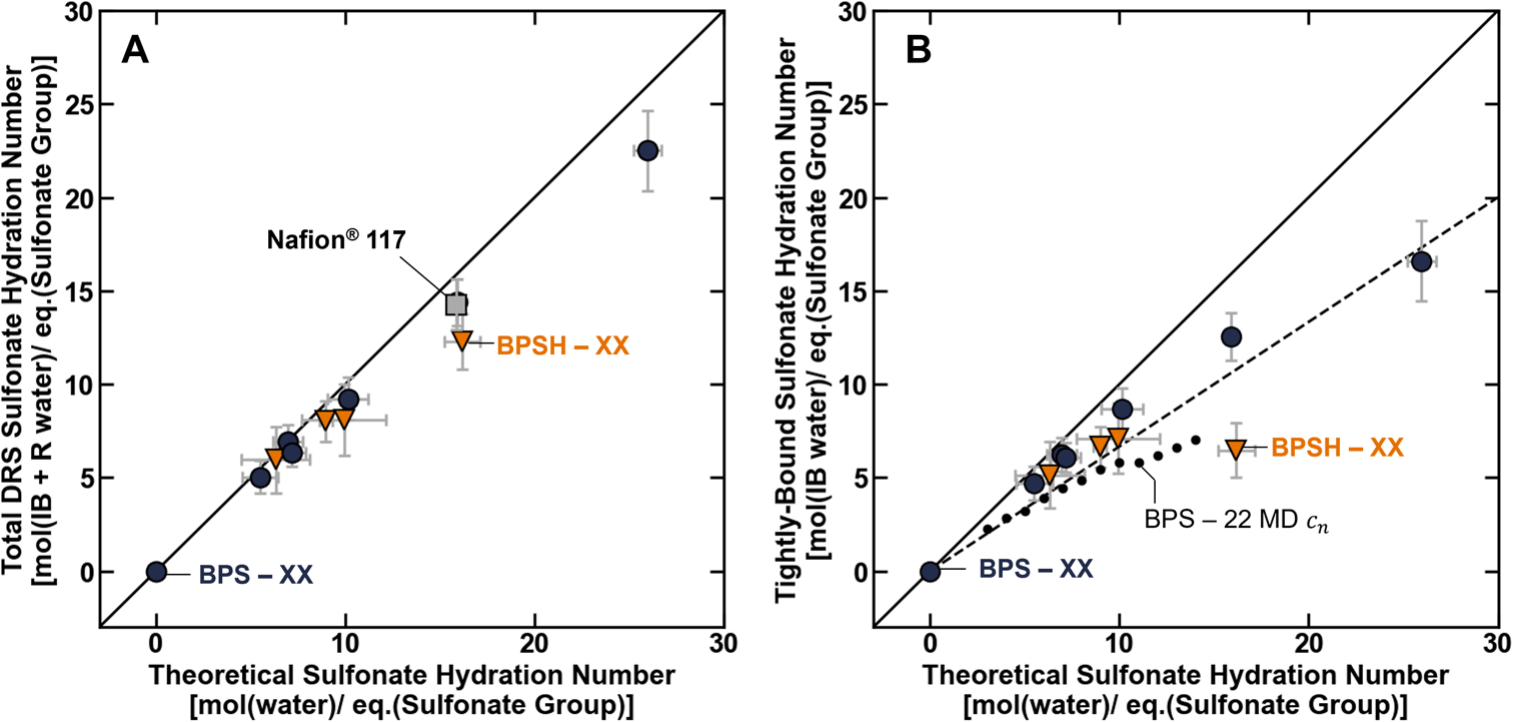
DRS measured (A) total sulfonate group hydration number and (B) irrotationally bound (i.e., tightly bound) sulfonate group hydration number for BPS—XX (blue ●), BPSH—XX (orange ▼), and Nafion (gray ■) plotted as a function of the theoretical sulfonate group hydration number. The empty points are plotted using the coordination numbers of BPS—22 polymers determined via molecular dynamics simulations as reported by Vondrasek et al.^40^ The solid line is plotted as the parity line where the DRS measured hydration number is equal to the theoretical sulfonate group hydration number, and the dashed line is plotted to guide the eye. All measurements were made at room temperature (i.e., 295 ± 1 K), and the uncertainty was propagated from the relative permittivity and water uptake measurements.

The number of water molecules in the primary hydration shell of the sulfonate groups can be estimated by calculating the irrotationally bound (or tightly bound) hydration number of the sulfonate groups as $\lambda_{SO_{3}^{-},IB}^{DRS}=c_{w,IB}^{m,ap}/c_{SO_{3}^{-}}^{m}$ (Figure 7B). While this tightly bound hydration number also scales with the theoretical hydration number, most of the data are below the parity line, suggesting that there is generally enough water in the polymer matrix to satisfy the minimum number of water molecules required to solvate the sulfonate groups (Figure 7B). Satisfyingly, the observed relationship between the experimentally measured tightly bound hydration number and theoretical hydration number is similar to the sulfonate group/water molecule coordination number for BPS—22 determined by molecular dynamics (MD) simulations as reported by Vondrasek et al. (Figure 7B).^40^ The agreement between the experimental data and the previously reported MD data helps validate the hydration numbers determined by DRS, which supports the use of this technique to estimate the effective charge group hydration in sulfonated polymers. The intermaterial differences observed in the tightly bound hydration number are consistent with the scaling relationships observed for the tightly bound water molecule concentrations (i.e., there are fewer water molecules in the primary hydration shell of polymers with weaker sulfonic acids) (c.f. Figures 7B and 5A).

#### 3.1.3 Dielectric Constant

The low-frequency static permittivity, or dielectric constant, is an important material property that is related to polymer thermodynamic properties and is useful to model ion transport processes in hydrated polymers. The dielectric constant can be calculated using the parameters fit to the frequency-dependent relative permittivity as (Figure 1):

13 $$\varepsilon_{m}=\varepsilon_{\infty }+\sum \Delta \varepsilon_{j}$$

For each sulfonated polysulfone, the dielectric constant calculated from the relaxation model parameters was equal to the relative permittivity measured at 45 MHz, which was the low-frequency limit of the measurement (Figures 1 and 2).

The dielectric constant of the charged polymers increases with increasing polymer water volume fraction (Figure 8), which is consistent with observations made for uncharged polymers.^34^ However, distinct scaling relationships are observed between the water volume fraction and dielectric constant for BPS—XX, BPSH—XX, and Nafion, and at a given water volume fraction, the relative order of the dielectric constants is Nafion > BPSH—XX > BPS—XX (Figure 8). This result makes sense because the ordering of the dielectric constants is the same as the ordering of the concentration of rotationally bound and bulk-like water molecules (Figure 5B,C). The water molecules in these states can contribute to dielectric relaxation processes (i.e., polarize in the presence of an electromagnetic field), so the observed increase in the dielectric constant corresponds to the increase of these polarizable water concentrations in the charged polymers. These observations suggest that the dielectric constant of the sulfonated polymers can effectively be modified through chemical modifications and pretreatment techniques that influence the strength of the sulfonic acid/conjugate base pair tethered to the polymer and size of the hydrated domain.

**Figure 8:**
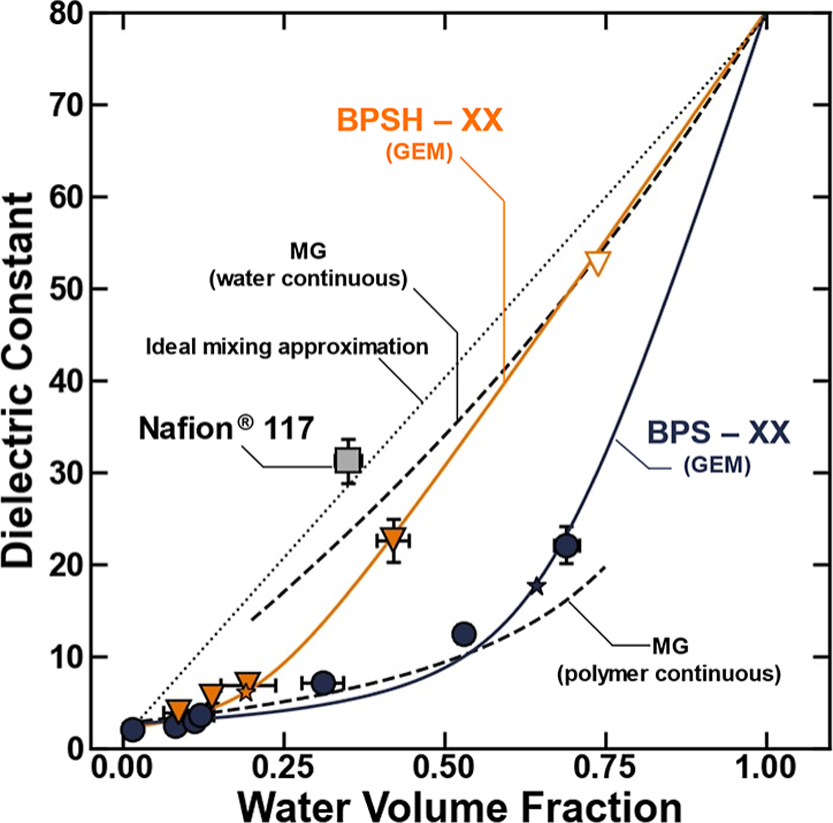
Dielectric constant of BPSH—XX (orange ▼), BPS—XX (blue ●), and Nafion (gray ■) polymers plotted as a function of water volume fraction in the hydrated polymer. The dashed lines are calculated using the Maxwell Garnett (MG) model, the solid lines are calculated by applying the general effective medium (GEM) approximation to the data, and the dotted line is plotted as a linear volumetric approximation of the dielectric constant that assumes an ideal mixture of polymer and water.^41^ The stars are plotted to represent the critical water volume fraction (i.e., percolation threshold) fit to the experimental data in the application of the GEM model. The uncertainties were taken as the standard deviation from the mean of three measurements.

The dielectric constant can be used in thermodynamic models that describe ion transport in hydrated polymers, and as a result, there have been significant efforts to model the polymer dielectric constant as a function of polymer physical properties (e.g., water volume fraction) (Figure 8). In the simplest case, a hydrated polymer can be approximated as a heterogeneous mixture of water and dry polymer, so many modeling efforts employ heterogeneous phase models that describe the properties of the mixture using the properties of the pure components.^14,41,42^ The simplest of these models assumes a linear relationship that connects the dielectric constant of the dry polymer with that of pure water,^41^ and this simple model works well for hydrated Nafion (Figure 8).^41^ The ability of the linear approximation to describe the dielectric constant properties of Nafion is likely due to the phase-segregated morphology of Nafion and the observation that water/polymer interactions do not prevent water molecules from polarizing in the presence of an electromagnetic field (Figure 6). In other words, Nafion may represent an ideal mixture where water/polymer interactions do not appreciably influence the polarizability of water molecules in the polymer, and the overall dielectric constant is governed essentially by the concentration of water in the polymer (Figure 8).

However, many hydrated polymers are not ideal mixtures of water and polymer, and in many systems, water/polymer interactions influence the ability of water molecules to polarize in an electromagnetic field (e.g., these sulfonated polysulfones).^17,34^ Under these circumstances, the linear approximation grossly overpredicts the dielectric constant of hydrated polymers (Figure 8).^15,17,34^ Previously, the Maxwell–Garnett (MG) model, which is another heterogeneous phase model, has been used to describe the relationship between water content and dielectric constant in hydrated uncharged polymers.^14,18,42,43^ An advantage of the MG model is that it requires no adjustable parameters and is theoretically exact in the low-water volume fraction limit.^43^ However, a limitation of the MG model is that it requires an assumption of a continuous phase (i.e., a polymer-continuous material or water-continuous material), and this assumption results in poor predictions of material properties near the critical volume fraction where the material transitions between polymer and water continuity (Figure 8).^43^ This critical water volume fraction, φ_c_, is commonly referred to as the percolation threshold.^43,44^ Hydrated sulfonated polysulfones exhibit a percolation transition with increasing degree disulfonation,^10,11,28,31^ and the different scaling relationships observed between the dielectric constant and water volume fraction of the sulfonated polysulfones may be driven by differences in the percolation threshold in acidified and nonacidified polymers (i.e., BPSH—XX breaks from the polymer continuous MG model at lower water volume fractions than BPS—XX). These differences in acidified and nonacidified sulfonated polysulfone have been observed previously.

Effective medium models can be used to describe the dielectric properties of heterogeneous materials that exhibit a percolation threshold.^43^ The most common application of the effective medium model is the Bruggeman effective medium model (BEM), which predicts a percolation threshold at φ_w_ = 0.3, but the general effective medium (GEM) model can be used to describe the dielectric properties of heterogeneous materials that exhibit percolation thresholds at other water volume fractions^45,46^

14 $$\left(1-\varphi_{w}\right)\frac{\varepsilon_{p}^{1/s}-\varepsilon_{m}^{1/s}}{\varepsilon_{p}^{1/s}-A\varepsilon_{m}^{1/s}}+\varphi_{w}\frac{\varepsilon_{w}^{1/t}-\varepsilon_{m}^{1/t}}{\varepsilon_{w}^{1/t}-A\varepsilon_{m}^{1/t}}=0$$

where ε_p_ and ε_w_ are the dielectric constants of the homogeneous components in the mixture (i.e., dry polymer and water), and φ_w_ is the polymer water volume fraction. The parameter *A* in the GEM is related to the critical water volume fraction as *A* = (1 – φ_c_)/φ_c_, and the exponents *s* and *t* depend on the geometry of the system. For φ_c_ = 0.3 and *s* = *t* = 1, the GEM reduces to the BEM.^45,46^

This GEM model was applied to the experimental data to quantitatively determine the critical volume fraction (i.e., percolation threshold) for the BPS—XX and BPSH—XX polymers (Figure 8). Reasonable fits for both polymers were obtained using *s* = *t* = 1 and a dry polymer dielectric constant of 2.67, which has been used previously.^14^ The dielectric constant of water was 78.^34^ The GEM model generally described all the data within experimental error and supported previously reported observations in sulfonated polysulfone where the percolation threshold occurs at lower water volume fractions for BPSH—XX than for BPS—XX.^28^ These values of the critical water volume fraction were determined to be 0.19 for BPSH—XX and 0.64 for BPS—XX (Figure 8).

Models that connect polymer dielectric constant properties, with other physical properties, such as fixed charge concentration, would also be helpful to fully describe the thermodynamic environment (i.e., ionic strength and dielectric constant) in hydrated polymers. In aqueous solutions, such as those containing NaCl, the dielectric constant decreases with increasing ionic strength (i.e., increased charge concentration) due to a phenomenon referred to as dielectric saturation (Figure 9).^35,47,48^ Dielectric saturation effectively describes how interactions between water molecules and charges interfere with the ability of water molecules to polarize in the presence of an external electromagnetic field.^47,49^ The dielectric constant of the charged polymers decreases with increasing sulfonate concentration, suggesting that, to a first approximation, dielectric saturation effects contribute to the relationship between the dielectric constant and charge strength in these sulfonated polysulfones (Figure 9). At a given fixed charge concentration, the relative ordering of the dielectric constant is Nafion > BPSH—XX > BPS—XX (i.e., scales inversely with the strength of the conjugate base), which is consistent with the previously described physical picture where, at a given fixed charge concentration, the most densely charged sulfonate groups suppress the dielectric constant of the hydrated polymers to the greatest extent (Figure 9). Interestingly, this results in the dielectric constant of Nafion being approximately equal to that of a NaCl solution at a comparable charge density (Figure 9), which may suggest that the perfluorinated sulfonate groups in Nafion have similar interactions with water molecules as NaCl.

**Figure 9:**
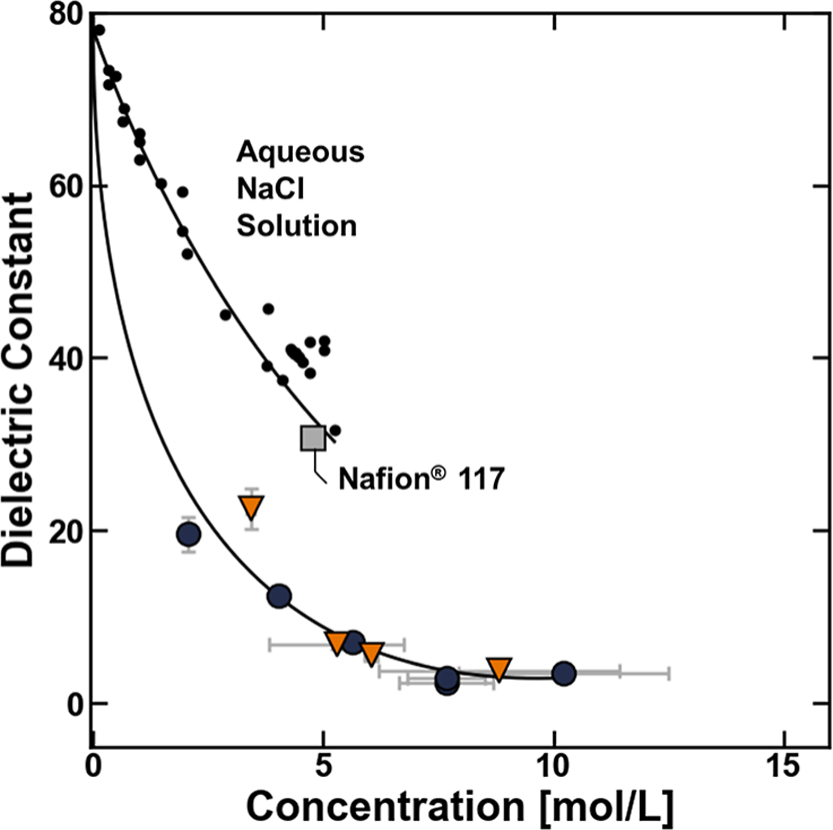
Dielectric constant of BPSH—XX (orange ▼), BPS—XX (blue ●), and Nafion (gray ■), plotted as a function of their sulfonate (or fixed charge) group concentration in the thermodynamic units basis (i.e., [mol/L (water sorbed)]). The dielectric constants of aqueous NaCl solutions^48^ are plotted as a function of their NaCl concentration.^48^ The solid lines are calculated using the empirical correlation model. The uncertainties were taken as the standard deviation from the mean of three measurements.

The relationship between the concentration of an arbitrary charge, i, and the dielectric constant can be quantitatively described using an empirical correlation model of the form:^47^

15 $$\varepsilon_{r}\left(c_{i}\right)=\varepsilon_{w}+\sum_{j=1}^{5}\theta_{j}^{EC}c_{i}^{j/2}$$

where ε_r_ is the relative permittivity (i.e., dielectric constant), ε_w_ is the dielectric constant of pure water, θ^EC^ is an empirical parameter, and *c*_i_ is the concentration of the charge. This empirical correlation model generally describes the concentration dependence of the relative permittivity in aqueous electrolyte solutions such as NaCl, albeit with reasonable scatter (Figure 9) and is useful in models that describe ionic interactions in solution.^47^ This empirical correlation describes the relative permittivity of the sulfonated polysulfones with similar amounts of scatter as for NaCl solution (Figure 9), and in the subsequent section, we will test the ability of the empirical correlation model to describe the relationship between polymer charge concentration and the dielectric constant in a thermodynamic model that describes salt partitioning in sulfonated polysulfone.

### 3.2 Connecting Relative Permittivity and Salt Transport Properties

Previously, Xie et al. reported the fundamental salt transport properties of BPS—XX and BPSH—XX for their use as potential desalination materials, and generally, Xie et al. suggested that the overall salt permeability of the sulfonated polysulfones follow classic scaling relationships where salt permeability coefficients increase with polymer water content (i.e., *P*_s_ ∼ IEC (Figure 10)).^24^ In our measurements, we observed similar scaling relationships between salt permeability and water content in sulfonated polysulfone and Nafion (Figure 10). A key takeaway from Xie et al.’s fundamental study was that the salt permeability coefficient of the charged sulfonated polysulfones was generally smaller than the salt permeability for methacrylate-based hydrogels reported by Yasuda et al. when compared at comparable water contents (i.e., the BPS—XX and BPSH—XX polymers generally bracket the lower end of Yasuda et al.’s salt permeability data (Figure 10)).^24,50^

**Figure 10:**
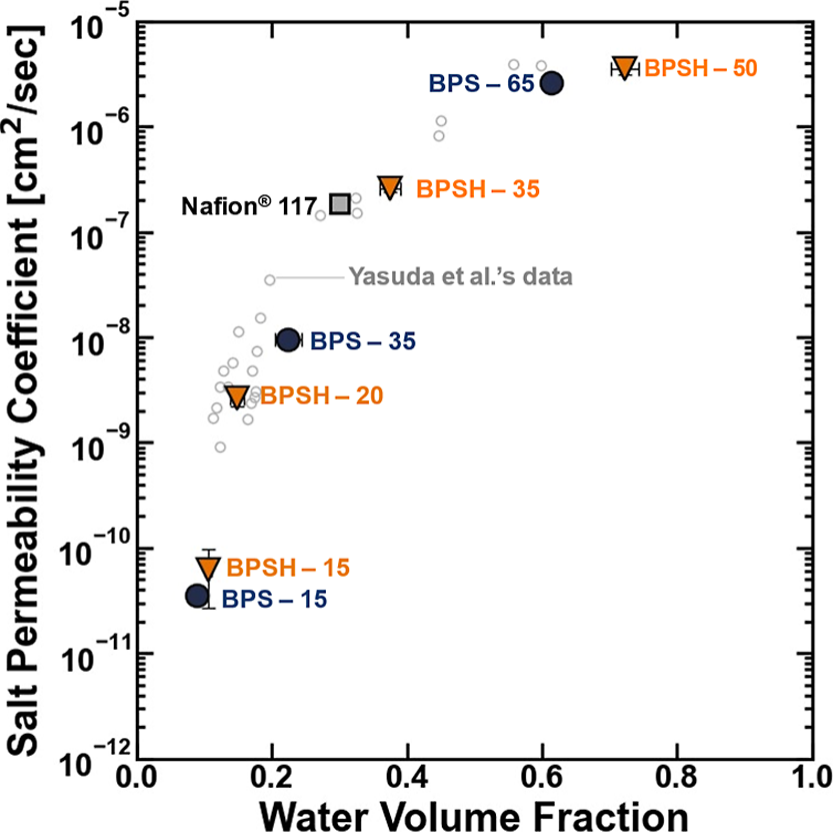
Salt permeability of BPSH—XX (orange ▼), BPS—XX (blue ●), Nafion (gray ■), and a series of model methacrylate based-hydrogels reported by Yasuda et al.^50^ (◯) plotted as a function of their water content. The uncertainties were taken as the standard deviation from the mean of three measurements.

Xie et al. reported that the salt permeability coefficient in the charged polymers was suppressed, relative to the uncharged polymers, primarily because of thermodynamic (i.e., sorption) factors.^24^ Similarly, we also found that the salt sorption coefficient of the sulfonated polymers was significantly reduced relative to that of the uncharged polymers (Figure 11). Observations of reduced salt sorption in charged polymers relative to uncharged polymers are often explained via a phenomenon referred to as Donnan exclusion, where the incorporation of fixed charges in the polymer effectively reduces the equilibrium concentration of mobile salt that partitions in the polymer matrix due to ion/fixed charge interaction effects.^25,51^ While the Donnan exclusion mechanism qualitatively describes the observations that salt sorption is suppressed in the charged polymers relative to uncharged polymers, other exclusion mechanisms, such as dielectric exclusion, which is related to the polymer-relative permittivity properties, can contribute to partitioning phenomenon in charged and uncharged polymers as well.^25,42,44,52^

**Figure 11:**
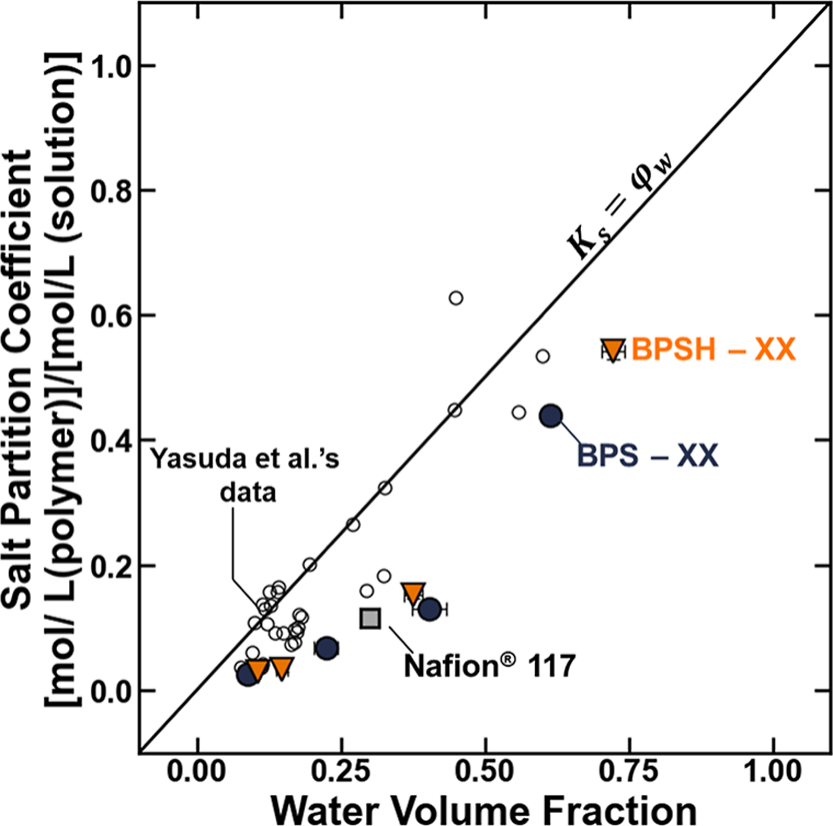
Salt partition coefficients for BPSH—XX (orange ▼), BPS—XX (blue ●), and Nafion (gray ■) and a series of model methacrylate-based hydrogels reported by Yasuda et al.^50^ (◯) reported as a function of their water content. The solid line represents an ideal case where the polymer salt sorption coefficient is equal to the water volume fraction. The uncertainties were taken as the standard deviation from the mean of three measurements.

Dielectric exclusion phenomena describe how ion solvation interactions contribute to partitioning properties in hydrated polymers.^25,41,42,44^ For salt to partition into a hydrated polymer, ions must be able to solvate (i.e., dissociate) in the polymer matrix, and charge dissociation is only possible if there are a sufficient number of polar molecules (i.e., water molecules) that can sufficiently stabilize the separation of ionic charges.^41^ For this reason, ions are less likely to dissociate in low dielectric constant materials where there are few water molecules available to polarize and stabilize ionic charge separation, and the salt partition coefficient of low dielectric constant polymers is often reduced compared to materials with larger dielectric constants.^15,17,34^

The observations in the preceding sections suggest that fixed charges can influence the ability of water molecules to polarize, and these irrotational binding interactions contribute to the dielectric saturation phenomenon that reduces the dielectric constant of charged polymers with increasing charge concentration (Figure 9). Under these circumstances, the observation that the partition coefficients of the sulfonated polymers are significantly smaller than those of uncharged polymers may also be consistent with a physical picture where incorporation of fixed charges in the polymer matrix suppresses the polymer dielectric constant, and the reduced polarizability of water molecules in highly charged polymers increases the ability of the dielectric exclusion mechanism to suppress salt sorption relative to uncharged polymers. This proposed physical picture is consistent with Freger’s recent perspective that many of the physical phenomena observed in hydrated polymers are incorrectly attributed to only Donnan exclusion interactions, where in many of these cases, dielectric exclusion plays a significant role as well.^44^ Understanding how dielectric saturation contributes to ion sorption in charged polymers would be helpful to develop molecular engineering strategies for charged polymers.

#### 3.2.1 Accounting for Dielectric Saturation When Modeling Salt Sorption

Donnan Theory, which describes the equilibrium between a charged polymer and an aqueous electrolyte, can be used as a framework to understand how different interactions contribute to the salt partition coefficients of charged polymers.^51^ This theory is derived only from considering the equilibrium condition between a charged polymer and solution (i.e., minimization of the Gibbs free energy between the two phases at equilibrium) and electronuetrality and defines the partition coefficient, *K*_s_, as^25^

16 $$K_{s}={\left[\frac{1}{4}{\left(\frac{C_{A}^{m}}{C_{s}^{s}}\right)}^{2}+{\left(\frac{\gamma_{\pm }^{s}}{\gamma_{\pm }^{m}}\right)}^{2}\right]}^{1/2}-\frac{1}{2}\frac{C_{A}^{m}}{C_{s}^{s}}$$

where *C*_A_^m^ is the concentration of fixed charges in the polymer, γ_±_^s^ and γ_±_^m^ are the mean ionic activity coefficients in the solution and polymer, and *C*_s_^s^ is the concentration of salt in the external solution. In the simplest case where the difference (between solution and polymer) in thermodynamic ionic nonideality is negligible (e.g., $\frac{\gamma_{\pm }^{s}}{\gamma_{\pm }^{m}}=1$ ), the theory is referred to as the Ideal Donnan theory, which suggests that the salt partition coefficient scales as $K_{s}∼\frac{C_{s}^{s}}{C_{A}^{m}}$ ^53^ This scaling relationship is the basis for Donnan exclusion because as *C*_A_^m^ increases, the partition coefficient is expected to decrease (Figure 12A). Indeed, the salt partitioning properties of the charged polymers characterized in this study qualitatively obey the Ideal Donnan scaling relationship (Figure 12A), but as discussed previously, this observed scaling relationship may also be consistent with a physical picture in which salt sorption is suppressed by dielectric saturation with increasing fixed charge concentration, and it is unclear if this scaling relationship should be attributed to the Donnan exclusion mechanism or the dielectric exclusion mechanism.

**Figure 12:**
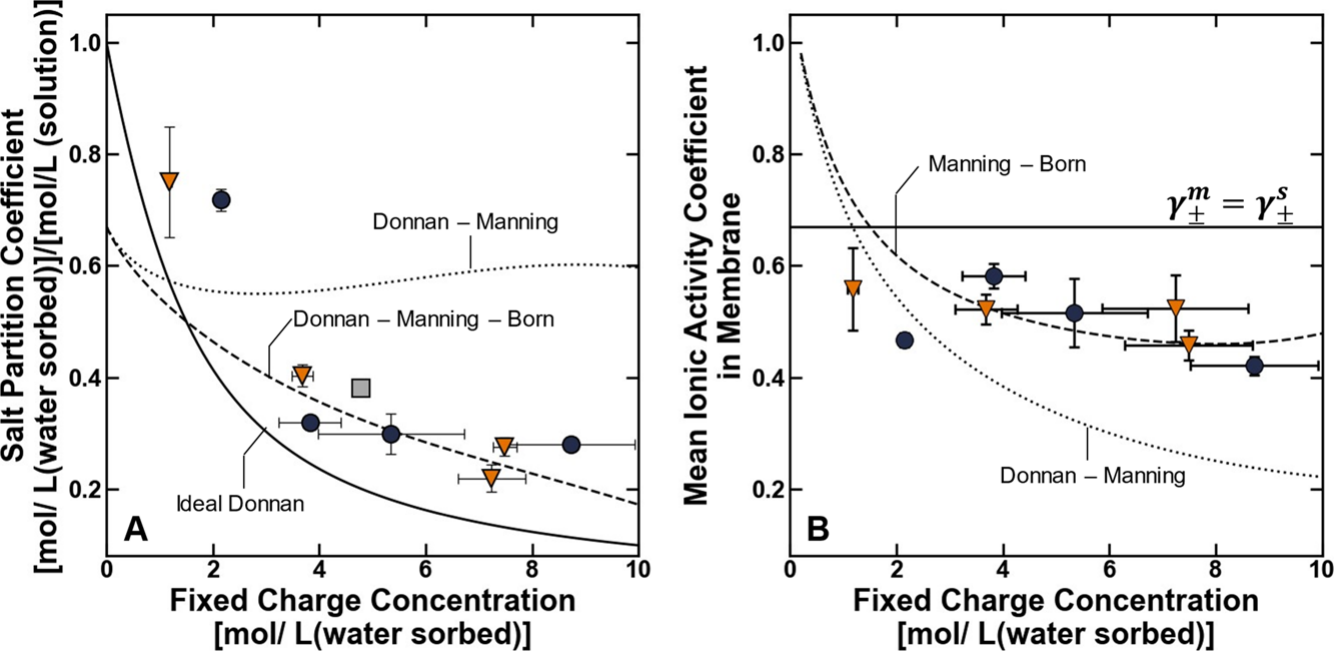
(A) Salt partition coefficient and (B) mean ionic activity coefficient of BPSH—XX (orange ▼), BPS—XX (blue ●), and Nafion reported as a function of their fixed charge concentration. The solid lines are calculated using the Ideal Donnan model (i.e., γ_±_^m^ = γ_±_^s^), the dashed lines represent the Donnan–Manning–Born model, and the dotted lines represent the Donnan–Manning model. The uncertainties were taken as the standard deviation from the mean of three measurements.

In most cases, the difference (between solution and polymer) in thermodynamic ionic nonideality cannot be ignored because charged polymers provide a different thermodynamic environment than aqueous electrolyte solutions (e.g., higher ionic strength and lower dielectric constants).^54,55^ Under these circumstances, the Donnan equation can be combined with thermodynamic models that describe the mean ionic activity coefficients in the hydrated polymer by accounting for the influence of specific ionic interactions on thermodynamic nonideality. The Donnan–Manning model is one such application that uses Manning’s counterion condensation model to describe the mean ionic activity coefficients by considering interactions between ions and fixed charges.^56^ On average, the interactions described by the Manning model deviate from ideality in the favorable direction because counterions with the opposite charge relative to the polymer fixed charges partition into the polymer more readily than the co-ions with the same charge relative to the polymer fixed charges, and interactions between counterions and fixed charges are thermodynamically favorable.^55^ The Donnan–Manning model provides accurate predictions of salt partitioning in many hydrated polymers suggesting that, to a first approximation, ion/fixed charge interactions govern thermodynamic nonideality in charged polymers.^23,54–59^

This Donnan–Manning model, however, generally overpredicts the extent of these favorable thermodynamic nonidealities in sulfonated polysulfone and as a result overpredicts the salt partition coefficients.^22,25^ This result is because the Manning model effectively suggests that reductions in the polymer dielectric constant increase the extent of thermodynamically favorable interactions in charged polymers by increasing the length scale over which the favorable electrostatic interactions between counterions and fixed charges persist, and this prediction contradicts the physical picture suggested by dielectric exclusion.^25^ Recently, we derived the Donnan–Manning–Born model by modifying the Manning model to account for dielectric exclusion interactions (i.e., incorporated the excess solvation energy in the Poisson–Boltzmann equation used to derive the Manning model), and this model accurately described salt partitioning in a series of hydroquinone- and methoxy hydroquinone-containing sulfonated polysulfones with fixed charge concentrations ranging from 4 to 6 mol/L (water sorbed) equilibrated with 0.5 M–4 M NaCl.^25^

In our previous study, the dielectric constant of the sulfonated polysulfone remained relatively constant over the range of external salt concentrations, which is perhaps consistent with a physical picture where, in the high concentration limit of Figure 9, added salt does not significantly increase the extent of dielectric saturation in the sulfonated polysulfones.^25^ Here, we applied the Donnan–Manning–Born model to the sulfonated polysulfone to describe the influence of modifications in polymer chemistry and the resulting variations in the thermodynamic environment (i.e., varied fixed charge concentration and dielectric constant), on polymer salt partitioning properties (Figure 12). We compared this model to the applications of the Donnan–Manning–Donnan and Ideal Donnan models for the sulfonated polysulfone to determine how different interactions contribute to polymer salt partitioning properties (Figure 12). For the sake of brevity, the model application is described in greater detail elsewhere,^25^ but effectively, the empirical correlation for the sulfonated polysulfone (eq 15) was used to model the variation in dielectric constant with polymer fixed charge concentration in the Manning–Born or Manning model, which was substituted into the Donnan equation to obtain an equation that was implicit in *C*_s_^m^. This equation was solved to determine *K*_s_ as a function of *C*_A_^m^ (Figure 9).

Generally, the Donnan–Manning model predicts that the salt partition coefficient is relatively independent of the polymer fixed charge concentration in sulfonated polysulfone (Figure 12A). This result is consistent with a physical picture where, as the fixed charge concentration increases and the dielectric constant decreases, the Manning model predicts that thermodynamic interactions in the polymer matrix become increasingly nonideal in the favorable direction (Figure 12B). In this model, these increasingly favorable interactions compete with Donnan exclusion interactions, so that the salt partition coefficient does not change appreciably with the fixed charge concentration (Figure 12A). As a result, the Donnan–Manning model generally overpredicts the partition coefficients of the sulfonated polysulfones and does not capture the influence of increased charge concentration and reduced dielectric constant on the salt partitioning properties of the polymers (Figure 12) (although, model predictions can be improved by neglecting the fixed charge concentration dependence of the dielectric constant).^25^

Accounting for dielectric exclusion improves the model agreement with the experimental data, and the Donnan–Manning–Born model describes most of the experimental data within the experimental uncertainty (Figure 12). In the Manning–Born model, accounting for dielectric exclusion, which is a thermodynamically nonfavorable interaction, increases the predicted activity coefficients (i.e., suggests thermodynamic interactions become more nonfavorable) relative to Manning model predictions (Figure 12B). These results are consistent with a physical picture where in the high fixed charge concentration limit, dielectric saturation phenomena contribute to reducing the favorability of ion solvation interactions, but overall, favorable ion/fixed charge interactions influence the thermodynamic nonideality to a greater extent than the nonfavorable dielectric exclusion interactions (Figure 12B). These results ultimately suggest that combinations of dielectric and Donnan–Manning exclusion influence the salt partitioning process in sulfonated polysulfone.

In the low fixed charge concentration limit, both the Donnan–Manning and Donnan–Manning–Born models overpredict the mean ionic activity coefficients (i.e., suggest that thermodynamic interactions in the polymer are less favorable than the experimental measurements) resulting in underpredictions of the salt partition coefficients (Figure 12). This result is consistent with a physical picture where both models suggest that, with low concentrations of fixed charges and high polymer dielectric constants, there are few ion/fixed charge interactions and thermodynamic interactions are ideal (Figure 12B). This observation may be consistent with a physical picture where other interactions besides those between ions and fixed charges (e.g., ion/ion interactions) contribute to thermodynamic nonideality in these polymers as well, and accounting for these interactions may be important to describe thermodynamic nonideality in less densely charged polymers.

Finally, it is interesting to note that despite having a significantly higher dielectric constant, the salt sorption properties and mean ionic activity coefficients of Nafion are comparable to those of the sulfonated polysulfones at similar fixed charge concentrations (Figure 12). This result may be consistent with a physical picture, where effectively the increased dielectric constant in Nafion contributes to reductions in both the favorable ion-fixed charge interactions and nonfavorable ion solvation interactions, and these competing interactions cause Nafion to experience similar salt partition coefficients to the sulfonated polysulfone despite the differences in dielectric constant (Figure 12A). Ultimately, this result is consistent with the observation that the mean ionic activity coefficients of all of the polymers are statistically indistinguishable over the range of dielectric constants and fixed charge concentrations (Figure 12B), which may also be related to the nature of the competing ion solvation and ion/fixed charge interactions. These results may suggest that engineering strategies to reduce the dielectric constant of densely charged polymers may not directly correspond to reduced salt partition coefficients but that dielectric exclusion still plays a significant role in determining the salt partitioning properties of charged polymers.

## 4 Conclusions

Dielectric relaxation spectroscopy can be used to characterize both the relative permittivity and the state of water in sulfonated polysulfones and Nafion. Here, using DRS, we determined that the concentrations of irrotationally bound and bulk-like water molecules in sulfonated polymers are governed largely by the charge density of the sulfonate group. We found that as the charge density of this sulfonate group increases, the concentration of bulk-like water decreases, and this change in the state of water corresponds to reductions in the polymer dielectric constant. These changes in the state of water also correspond to the state of water in the solvation shells of the sulfonate group, so that more densely charged sulfonate groups have higher concentrations of irrotationally bound water molecules in their solvation shells. The relationship between the dielectric constant and water volume fraction in the sulfonated polymers was described by the effective medium approximation, which is a heterogeneous phase model, whereas the dielectric constant of Nafion is well described by an ideal mixing model, and these results are consistent with a physical picture where water/polymer interactions do not influence significantly the ability of water molecules to polarize in Nafion.

The relative permittivity data were connected to polymer salt transport properties to probe the molecular underpinnings of previously determined structure–property relationships in sulfonated polysulfone. The dielectric constant data was correlated to the polymer fixed charge concentration, and this relationship was used to describe the thermodynamic environment of the hydrated polymers. This correlation suggested that as the polymer fixed charge concentration increased, the polymer dielectric constant decreased, and this change may suggest that salt sorption is reduced in charged polymers due to a combination of Donnan and dielectric exclusion mechanisms. Using a thermodynamic model that accounted for Donnan and dielectric exclusion interactions, the Donnan–Manning–Born model, and considering the variation of polymer fixed charge concentration and dielectric constant, we were able to describe the salt partitioning properties of the sulfonated polysulfones. These results support the idea that dielectric exclusion contributes to partitioning processes in charged polymers, and this understanding could inform future molecular engineering strategies to improve polymer transport properties for separation applications.

## Supplementary Material



## Notes

NMR and FT-IR characterization data for the polymers (PDF)

## References

[1] Kamcev J., Freeman B. D. (2016). Charged Polymer Membranes for Environmental/Energy
Applications. Annu. Rev. Chem. Biomol. Eng..

[2] Dlugolecki P., Nymeijer K., Metz S., Wessling M. (2008). Current Status of Ion
Exchange Membranes for Power Generation from Salinity Gradients. J. Membr. Sci..

[3] Ran J., Wu L., He Y., Yang Z., Wang Y., Jiang C., Ge L., Bakangura E., Xu T. (2017). Ion Exchange Membranes: New Developments
and Applications. J. Membr. Sci..

[4] Semiat R. (2008). Energy Issues
in Desalination Processes. Environ. Sci. Technol..

[5] Havelka J., Fárová H., Jiříček T., Kotala T., Kroupa J. (2019). Electrodialysis-Based
Zero Liquid
Discharge in Industrial Wastewater Treatment. Water Sci. Technol..

[6] Lufrano F., Squadrito G., Patti A., Passalacqua E. (2000). Sulfonated
Polysulfone as Promising Membranes for Polymer Electrolyte Fuel Cells. J. Appl. Polym. Sci..

[7] Park H. B., Freeman B. D., Zhang Z.-B., Sankir M., McGrath J. E. (2008). Highly
Chlorine-Tolerant Polymers for Desalination. Angew. Chem., Int. Ed..

[8] Geise G. M., Paul D. R., Freeman B. D. (2014). Fundamental Water and Salt Transport
Properties of Polymeric Materials. Prog. Polym.
Sci..

[9] Geise G. M., Lee H.-S., Miller D. J., Freeman B. D., McGrath J. E., Paul D. R. (2010). Water Purification
by Membranes: The Role of Polymer
Science. J. Polym. Sci., Part B: Polym. Phys..

[10] Roy A., Hickner M. A., Lee H.-S., Glass T., Paul M., Badami A., Riffle J. S., McGrath J. E. (2017). States of Water
in Proton Exchange Membranes: Part A - Influence of Chemical Structure
and Composition. Polymer.

[11] Kim Y. S., Dong L., Hickner M. A., Glass T. E., Webb V., McGrath J. E. (2003). State of Water in Disulfonated Poly­(Arylene
Ether Sulfone)
Copolymers and a Perfluorosulfonic Acid Copolymer (Nafion) and Its
Effect on Physical and Electrochemical Properties. Macromolecules.

[12] Smedley S. B., Chang Y., Bae C., Hickner M. A. (2015). Measuring Water
Hydrogen Bonding Distributions in Proton Exchange Membranes Using
Linear Fourier Transform Infrared Spectroscopy. Solid State Ionics.

[13] Chang K., Luo H., Geise G. M. (2019). Water Content,
Relative Permittivity, and Ion Sorption
Properties of Polymers for Membrane Desalination. J. Membr. Sci..

[14] Chang K., Luo H., Geise G. M. (2021). Influence
of Salt Concentration on Hydrated Polymer
Relative Permittivity and State of Water Properties. Macromolecules.

[15] Luo H., Chang K., Bahati K., Geise G. M. (2019). Functional Group
Configuration Influences Salt Transport in Desalination Membrane Materials. J. Membr. Sci..

[16] Luo H., Chang K., Bahati K., Geise G. M. (2019). Engineering Selective
Desalination Membranes via Molecular Control of Polymer Functional
Groups. Environ. Sci. Technol. Lett..

[17] Chang K., Geise G. M. (2020). Dielectric Permittivity
Properties of Hydrated Polymers:
Measurement and Connection to Ion Transport Properties. Ind. Eng. Chem. Res..

[18] Woodward, W. H. H. Broadband Dielectric Spectroscopy—A Practical Guide. In ACS Symposium Series; Woodward, W. H. H. , Ed.; American Chemical Society: Washington, DC, 2021; Vol. 1375, pp 3–59.10.1021/bk-2021-1375.ch001.

[19] Lu Z., Lanagan M., Manias E., Macdonald D. D. (2009). Two-Port
Transmission Line Technique for Dielectric Property Characterization
of Polymer Electrolyte Membranes. J. Phys. Chem.
B.

[20] Lu Z., Polizos G., Macdonald D. D., Manias E. (2008). State of Water in Perfluorosulfonic
Ionomer (Nafion 117) Proton Exchange Membranes. J. Electrochem. Soc..

[21] Paddison S. J., Bender G., Kreuer K.-D., Nicoloso N., Jr T. A. Z. (2000). The
Microwave Region of the Dielectric Spectrum of Hydrated Nafion®
and Other Sulfonated Membranes. J. New Mater.
Electrochem. Syst..

[22] Chang K., Luo H., Bannon S. M., Lin S. Y., Agata W. A. S., Geise G. M. (2021). Methoxy
Groups Increase Water and Decrease Salt Permeability Properties of
Sulfonated Polysulfone Desalination Membranes. J. Membr. Sci..

[23] Bannon S. M., Geise G. M. (2024). Influence of Donnan
and Dielectric Exclusion on Ion
Sorption in Sulfonated Polysulfones. J. Membr.
Sci..

[24] Xie W., Cook J., Park H. B., Freeman B. D., Lee C. H., McGrath J. E. (2011). Fundamental Salt
and Water Transport Properties in
Directly Copolymerized Disulfonated Poly­(Arylene Ether Sulfone) Random
Copolymers. Polymer.

[25] Bannon S. M., Geise G. M. (2024). Influence of Donnan
and Dielectric Exclusion on Ion
Sorption in Sulfonated Polysulfones. J. Membr.
Sci..

[26] Daryaei A., Miller G. C., Willey J., Roy Choudhury S., Vondrasek B., Kazerooni D., Burtner M. R., Mittelsteadt C., Lesko J. J., Riffle J. S., McGrath J. E. (2017). Synthesis and Membrane
Properties of Sulfonated Poly­(Arylene Ether Sulfone) Statistical Copolymers
for Electrolysis of Water: Influence of Meta- and Para-Substituted
Comonomers. ACS Appl. Mater. Interfaces.

[27] Wohlfarth A., Smiatek J., Kreuer K.-D., Takamuku S., Jannasch P., Maier J. (2015). Proton Dissociation
of Sulfonated Polysulfones: Influence of Molecular
Structure and Conformation. Macromolecules.

[28] Wang C., Paddison S. J. (2014). Mesoscale Modeling
of Hydrated Morphologies of Sulfonated
Polysulfone Ionomers. Soft Matter.

[29] Paul M., Park H. B., Freeman B. D., Roy A., McGrath J. E., Riffle J. S. (2008). Synthesis and Crosslinking of Partially
Disulfonated
Poly­(Arylene Ether Sulfone) Random Copolymers as Candidates for Chlorine
Resistant Reverse Osmosis Membranes. Polymer.

[30] Xie W., Ju H., Geise G. M., Freeman B. D., Mardel J. I., Hill A. J., McGrath J. E. (2011). Effect
of Free Volume on Water and Salt Transport Properties
in Directly Copolymerized Disulfonated Poly­(Arylene Ether Sulfone)
Random Copolymers. Macromolecules.

[31] Wang F., Hickner M., Kim Y. S., Zawodzinski T. A., McGrath J. E. (2002). Direct Polymerization of Sulfonated
Poly­(Arylene Ether
Sulfone) Random (Statistical) Copolymers: Candidates for New Proton
Exchange Membranes. J. Membr. Sci..

[32] Wang F., Hickner M., Ji Q., Harrison W., Mecham J., Zawodzinski T. A., McGrath J. E. (2001). Synthesis of Highly Sulfonated Poly­(Arylene
Ether Sulfone) Random (Statistical) Copolymers via Direct Polymerization. Macromol. Symp..

[33] McCormack P. M., Luo H., Geise G. M., Koenig G. M. (2020). Conductivity, Permeability, and Stability
Properties of Chemically Tailored Poly­(Phenylene Oxide) Membranes
for Li+ Conductive Non-Aqueous Redox Flow Battery Separators. J. Power Sources.

[34] Chang K., Luo H., Geise G. M. (2019). Water Content,
Relative Permittivity, and Ion Sorption
Properties of Polymers for Membrane Desalination. J. Membr. Sci..

[35] Buchner R., Hefter G. T., May P. M. (1999). Dielectric
Relaxation of Aqueous
NaCl Solutions. J. Phys. Chem. A.

[36] Xie W., Geise G. M., Freeman B. D., Lee C. H., McGrath J. E. (2012). Influence
of Processing History on Water and Salt Transport Properties of Disulfonated
Polysulfone Random Copolymers. Polymer.

[37] Kim Y. S., Hickner M. A., Dong L., Pivovar B. S., McGrath J. E. (2004). Sulfonated
Poly­(Arylene Ether Sulfone) Copolymer Proton Exchange Membranes: Composition
and Morphology Effects on the Methanol Permeability. J. Membr. Sci..

[38] Mauritz K. A., Moore R. B. (2004). State of Understanding of Nafion. Chem. Rev..

[39] Roy S., Dang L. X. (2015). Water Exchange Dynamics around H3O+ and OH–
Ions. Chem. Phys. Lett..

[40] Vondrasek B., Wen C., Cheng S., Riffle J. S., Lesko J. J. H. (2021). Hydration, Ion
Distribution, and Ionic Network Formation in Sulfonated Poly­(arylene
ether sulfones). Macromolecules.

[41] Zhang H., Geise G. M. (2016). Modeling the Water
Permeability and Water/Salt Selectivity
Tradeoff in Polymer Membranes. J. Membr. Sci..

[42] Bannon S. M., Geise G. M. (2024). Application of the
Born Model to Describe Salt Partitioning
in Hydrated Polymers. ACS Macro Lett..

[43] Bergman D. J. (1978). The Dielectric
Constant of a Composite Material—A Problem in Classical Physics. Phys. Rep..

[44] Freger V. (2023). Dielectric
Exclusion, an Éminence Grise. Adv. Colloid
Interface Sci..

[45] McLachlan D. S. (1987). An Equation
for the Conductivity of Binary Mixtures with Anisotropic Grain Structures. J. Phys. C Solid State Phys..

[46] Brosseau C. (2002). Generalized
Effective Medium Theory and Dielectric Relaxation in Particle-Filled
Polymeric Resins. J. Appl. Phys..

[47] Silva G. M., Liang X., Kontogeorgis G. M. (2023). How to
Account for the Concentration
Dependency of Relative Permittivity in the Debye–Hückel
and Born Equations. Fluid Phase Equilib..

[48] Maribo-Mogensen B., Kontogeorgis G. M., Thomsen K. (2013). Modeling of Dielectric
Properties
of Aqueous Salt Solutions with an Equation of State. J. Phys. Chem. B.

[49] Paspureddi A., Sharma M. M., Katz L. E. (2022). Effect of Dielectric Saturation on
Ion Activity Coefficients in Ion Exchange Membranes. ACS Omega.

[50] Yasuda H., Lamaze C. E., Ikenberry L. D. (1968). Permeability
of Solutes through Hydrated
Polymer Membranes. Part I. Diffusion of Sodium Chloride. Makromol. Chem..

[51] Helfferrich, F. Ion Exchange; McGraw-Hill: New York, 1962.

[52] Yaroshchuk A. E. (2000). Dielectric
Exclusion of Ions from Membranes. Adv. Colloid
Interface Sci..

[53] Geise G. M., Falcon L. P., Freeman B. D., Paul D. R. (2012). Sodium
Chloride
Sorption in Sulfonated Polymers for Membrane Applications. J. Membr. Sci..

[54] Galizia M., Manning G. S., Paul D. R., Freeman B. D. (2019). Ion Partitioning
between Brines and Ion Exchange Polymers. Polymer.

[55] Kamcev J., Paul D. R., Freeman B. D. (2015). Ion Activity
Coefficients in Ion
Exchange Polymers: Applicability of Manning’s Counterion Condensation
Theory. Macromolecules.

[56] Kamcev J., Galizia M., Benedetti F. M., Jang E.-S., Paul D. R., Freeman B. D., Manning G. S. (2016). Partitioning
of Mobile Ions between
Ion Exchange Polymers and Aqueous Salt Solutions: Importance of Counter-Ion
Condensation. Phys. Chem. Chem. Phys..

[57] Kitto D., Kamcev J. (2022). Manning Condensation
in Ion Exchange Membranes: A Review
on Ion Partitioning and Diffusion Models. J.
Polym. Sci..

[58] Sujanani R., Katz L. E., Paul D. R., Freeman B. D. (2021). Aqueous Ion Partitioning
in Nafion: Applicability of Manning’s Counter-Ion Condensation
Theory. J. Membr. Sci..

[59] Sujanani R., Nordness O., Miranda A., Katz L. E., Brennecke J. F., Freeman B. D. (2023). Accounting for Ion Pairing Effects on Sulfate Salt
Sorption in Cation Exchange Membranes. J. Phys.
Chem. B.

