# Supplementary material for: Characterizing Hydrated Polymers via Dielectric Relaxation Spectroscopy: Connecting Relative Permittivity, State of Water, and Salt Transport Properties of Sulfonated Polysulfones
Source: Macromolecules. 2025 Jul 25;58(15):8271–87. doi: 10.1021/acs.macromol.5c00616 (PMC12356068; doi:10.1021/acs.macromol.5c00616)
Supplement: Supplementary file 1 [file ma5c00616_si_001.pdf]

Supporting Information

Characterizing Hydrated Polymers via Dielectric Relaxation Spectroscopy: Connecting Relative Permittivity, State of Water, and Salt Transport Properties of Sulfonated Polysulfones

Sean M. Bannon[1], Beatrice M. Tremblay[1,2,3], Andrew Boudreau[2], Nurshaun Sreedhar[2,4], Caroline Morin[1], Charles R. Leroux[1], Phu Phan[1], Abishek Roy[2], Mou Paul[2,], and Geoffrey M. Geise[1,*]*

To whom correspondence should be addressed: geise@virginia.edu

[1]Department of Chemical Engineering, University of Virginia,

Charlottesville, VA 22903 USA

[2]National Renewable Energy Laboratory,

Golden, CO 80401 USA

[3]Department of Chemical Engineering, University of California Santa Barbara,

Santa Barbara, CA 93106 USA

[4]Maseeh Department of Civil, Architectural and Environmental Engineering, The University of Texas at Austin, Austin, TX 78712 USA

Section S1. Structural verification of the polymers

The successful incorporation of sulfonate groups into the polymers was confirmed by FT-IR spectroscopy (Figure S1). Strong characteristic peaks at 1030 and 1098 cm^{-1} were assigned to symmetric and asymmetric stretching of the sulfonate groups, and these peaks were observed for all sulfonated copolymers from BPS-10 to BPS-65 (Figure S1). The absorbance intensities of these two characteristic peaks increased with higher SDCDPS content (Figure S1).

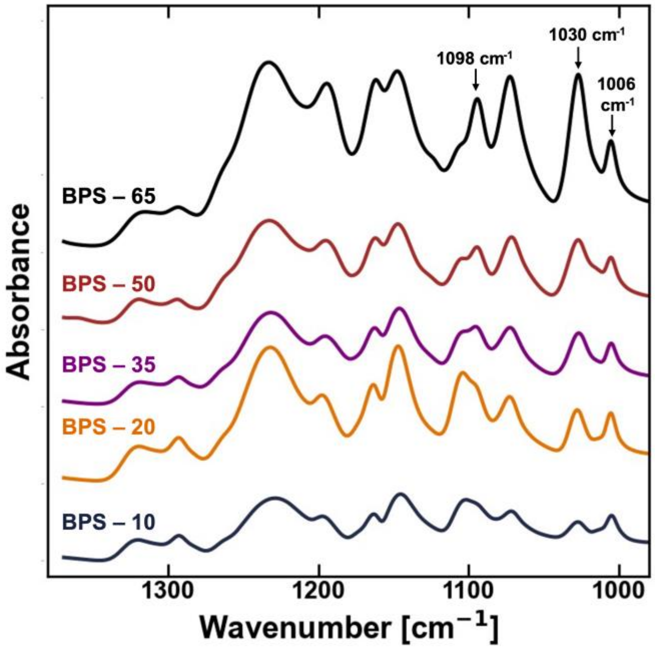

Figure S1. FTIR spectra for the BPS – XX polymers as a function of disulfonation.

The symmetric stretching of sulfonate groups at 1030 cm^{-1} can be compared to internal standards, such as the in-chain diphenyl ether absorption at 1006 cm^{-1}. Figure S2 provides quantitative analysis of the FT-IR data where a linear correlation was observed when the degree of disulfonation was plotted against the ratio of the two absorbances from the sulfonate groups (1030

cm^{-1}) and the diphenyl ether groups (1006 cm^{-1}). These results suggest that sulfonate groups were incorporated into the polymer in accordance with previous literature references.[1]

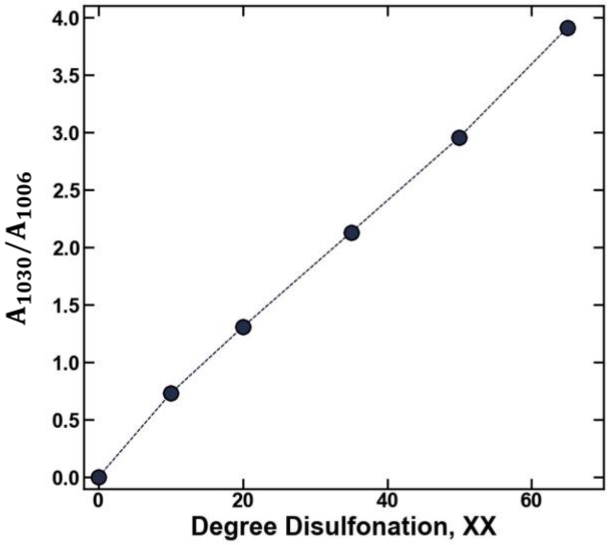

Figure S2. FT-IR correlation of degree of disulfonation with the ratio of the two absorbances from the sulfonate groups (1030 cm^{-1}), A_{1030}, and the diphenyl ether groups (1006 cm^{-1}), A_{1006}.

Proton NMR spectra also confirmed the sulfonated monomer inclusion in the polymer, and the degree of disulfonation (DS) can be calculated from ^{1}H NMR spectra. Figure S3 shows the proton NMR spectra of the BPS polymers as a function of the degree of disulfonation. The degree of disulfonation was confirmed from the ratio of the integrals of peaks at 7.025 ppm (e), 7.88 ppm (f) and 8.33 ppm (g), which are attributed to the sulfonated repeat unit to the protons at 7.95 ppm (k) in the un-sulfonated repeat unit as:

$$DS = \frac{(e + f + g)/3}{(e + f + g)/3 + k/2} \qquad \text{Eq. S1}$$

The degree of disulfonation, as calculated from Eq. S1, were used to calculate the ion exchange capacities (IECs) of the copolymers. Generally, the experimental IEC of the polymers obtained from the ^{1}H NMR spectroscopy data are in reasonable agreement with their theoretical values calculated from the ratio of co-monomers incorporated in the synthetic mixture (all experimental IECs were within approximately 5% of the theoretical IECs except for the BPS – 10, which was within approximately 25% of the theoretical IEC) (Table S1).

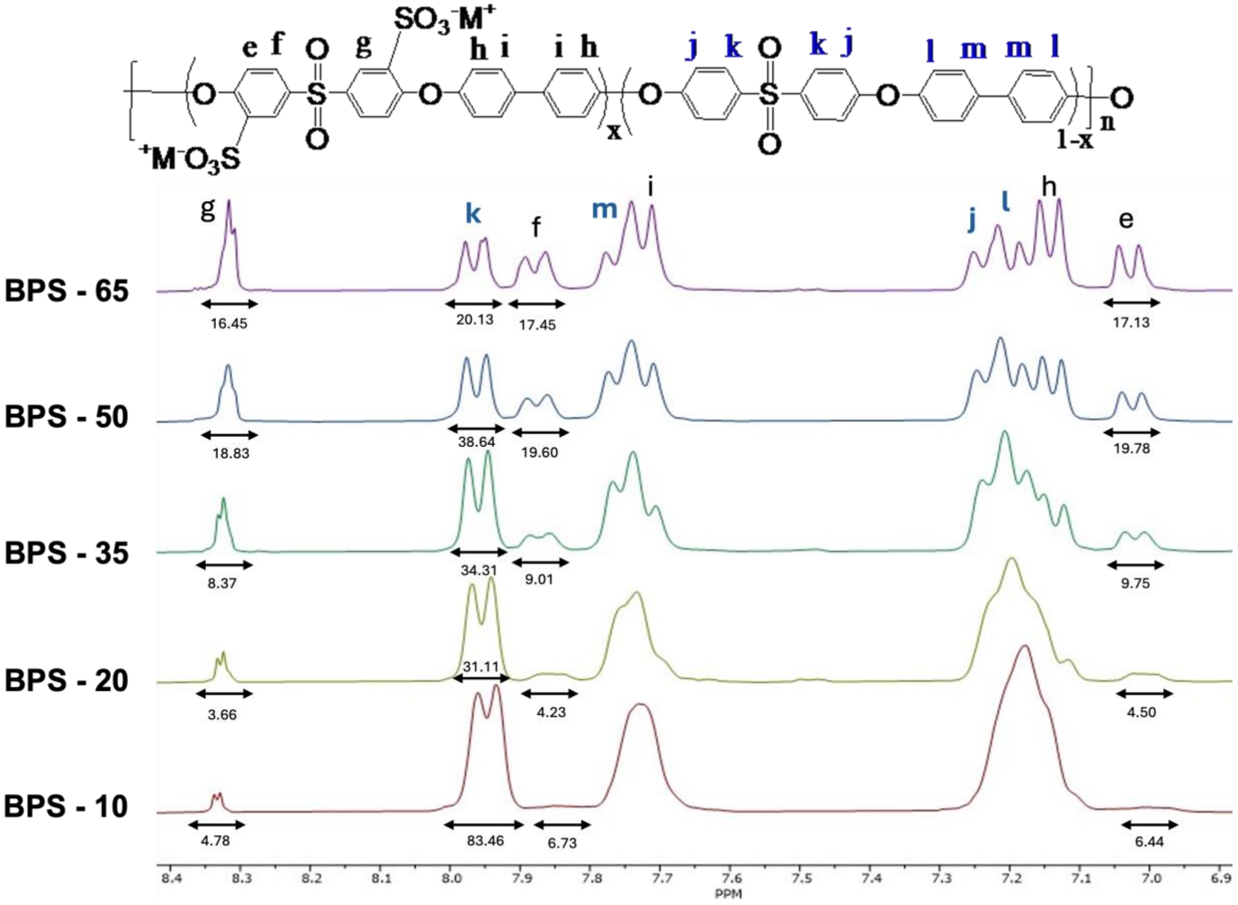

Figure S3. ^{1}H NMR spectra for the BPS – XX polymers as a function of disulfonation.

Table S1. Values of the ^{1}H NMR peak integrals obtained for the BPS – XX polymers and the corresponding NMR-determined degree of disulfonation and ion exchange capacity.

**Polymer**	**Peak e** ~6.95 ppm	**Peak f** ~7.8 ppm	**Peak g** ~8.25 ppm	**Peak k** ~ 7.9 ppm	**Degree of disulfonation**	**IEC from NMR (meq/g)**	**Theoretical IEC (meq/g)**
BPS – 10	6.44	6.73	4.78	83.46	0.13	0.60	0.48
BPS – 20	4.5	4.23	3.66	31.11	0.21	0.95	0.91
BPS – 35	9.75	9.01	8.37	34.31	0.35	1.46	1.48
BPS – 50	19.78	19.6	18.83	38.64	0.50	1.99	1.99
BPS – 65	17.13	17.45	16.45	20.13	0.63	2.36	2.44

S2. References

(1) Wang, F.; Hickner, M.; Kim, Y. S.; Zawodzinski, T. A.; McGrath, J. E. Direct Polymerization of Sulfonated Poly(Arylene Ether Sulfone) Random (Statistical) Copolymers: Candidates for New Proton Exchange Membranes. *Journal of Membrane Science* **2002**, *197* (1–2), 231–242. https://doi.org/10.1016/S0376-7388(01)00620-2.
